# Sex Steroids Induce Membrane Stress Responses and Virulence Properties in Pseudomonas aeruginosa

**DOI:** 10.1128/mBio.01774-20

**Published:** 2020-09-29

**Authors:** Celine Vidaillac, Valerie Fei Lee Yong, Marie-Stephanie Aschtgen, Jing Qu, Shuowei Yang, Guangfu Xu, Zi Jing Seng, Alexandra C. Brown, Md Khadem Ali, Tavleen K. Jaggi, Jagadish Sankaran, Yong Hwee Foo, Francesco Righetti, Anu Maashaa Nedumaran, Micheál Mac Aogáin, Dan Roizman, Jean-Alexandre Richard, Thomas R. Rogers, Masanori Toyofuku, Dahai Luo, Edmund Loh, Thorsten Wohland, Bertrand Czarny, Jay C. Horvat, Philip M. Hansbro, Liang Yang, Liang Li, Staffan Normark, Birgitta Henriques Normark, Sanjay H. Chotirmall

**Affiliations:** a Lee Kong Chian School of Medicine, Nanyang Technological University, Singapore; b Singapore Centre for Environmental Life Sciences Engineering, Nanyang Technological University, Singapore; c Department of Microbiology, Tumor and Cell Biology, Karolinska Institutet and Karolinska University Hospital Solna, Stockholm, Sweden; d Shenzhen Institutes of Advanced Technology, Chinese Academy of Sciences, Shenzhen, Guangdong, China; e Department of Otolaryngology, The Fifth Affiliated Hospital of Sun Yat-Sen University, Zhuhai, Guangdong, China; f Priority Research Centre for Healthy Lungs, Hunter Medical Research Institute and School of Biomedical Sciences and Pharmacy, University of Newcastle, Newcastle, New South Wales, Australia; g Department of Biological Sciences, National University of Singapore, Singapore; h Centre for Bio-Imaging Sciences, National University of Singapore, Singapore; i School of Materials Science and Engineering (MSE), Nanyang Technological University, Singapore; j Functional Molecules and Polymers, Institute of Chemical and Engineering Sciences, ICES, Agency for Science, Technology, and Research, A*STAR, Singapore; k Department of Clinical Microbiology, Trinity College Dublin, St. James’s Hospital Campus, Dublin, Ireland; l Faculty of Life and Environmental Sciences, University of Tsukuba, Tsukuba, Japan; m Centre for Inflammation, Centenary Institute, Sydney, NSW, Australia; n University of Technology Sydney, School of Life Sciences, Faculty of Science, Ultimo, NSW, Australia; University of Texas Southwestern Medical Center Dallas

**Keywords:** hormones, steroids, *Pseudomonas aeruginosa*, membrane stress, gender

## Abstract

Estrogen, a major female sex steroid hormone, has been shown to promote the selection of mucoid Pseudomonas aeruginosa in the airways of patients with chronic respiratory diseases, including cystic fibrosis. This results in long-term persistence, poorer clinical outcomes, and limited therapeutic options. In this study, we demonstrate that at physiological concentrations, sex steroids, including testosterone and estriol, induce membrane stress responses in P. aeruginosa. This is characterized by increased virulence and consequent inflammation and release of proinflammatory outer membrane vesicles promoting *in vivo* persistence of the bacteria. The steroid-induced P. aeruginosa response correlates with the molecular polarity of the hormones and membrane fluidic properties of the bacteria. This novel mechanism of interaction between sex steroids and P. aeruginosa explicates the reported increased disease severity observed in females with cystic fibrosis and provides evidence for the therapeutic potential of the modulation of sex steroids to achieve better clinical outcomes in patients with hormone-responsive strains.

## Abstract (precis)

Molecular mechanisms by which sex steroids interact with P. aeruginosa to modulate its virulence have yet to be reported. Our work provides the first characterization of a steroid-induced membrane stress mechanism promoting P. aeruginosa virulence, which includes the release of proinflammatory outer membrane vesicles, resulting in inflammation, host tissue damage, and reduced bacterial clearance. We further demonstrate that at nanomolar (physiological) concentrations, male and female sex steroids promote virulence in clinical strains of P. aeruginosa based on their dynamic membrane fluidic properties. This work provides, for the first-time, mechanistic insight to better understand and predict the P. aeruginosa related response to sex steroids and explain the interindividual patient variability observed in respiratory diseases such as cystic fibrosis that are complicated by gender differences and chronic P. aeruginosa infection.

## INTRODUCTION

Clinical observations and research reveal that males are at higher risk for bacterial infection; however, females are more prone to develop more severe illness with poorer outcomes, particularly at reproductive age (1–3). Genetic, anatomical, social, and behavioral differences have all been advanced to explain these gender biases, however, more recently, variations in the host innate and adaptive immune responses under the control of sex steroid hormones have been proposed (4–6). Estrogens exert immuno-protection by promoting natural killer cell activity and inducing the production of proinflammatory cytokines, including tumor necrosis factor alpha (TNF-α), interleukin-1 (IL-1), IL-6. IL-17, and IL-23, while testosterone exerts an opposing immunosuppressive effect (5). The regulation of immune responses by sex steroid hormones may at least partially explain differences in disease susceptibility; however, it does not fully explain the observed differences in disease severity and, importantly, microbial adaptation that occur between genders in the context of chronic pulmonary infection (7).

The last 2 decades have witnessed an exponential rise in the number of studies investigating the role of sex steroids on the progression of chronic infections (7–13). Many bacterial pathogens sense and respond to mammalian hormones, including sex steroids (5, 8, 13, 14). Nanomolar physiological concentrations of 17β-estradiol and estriol promote Pseudomonas aeruginosa alginate and pyocyanin production, surface motility, biofilm formation, and adherence to lung epithelia (8, 12). In contrast, micromolar supraphysiological concentrations of 17β-estradiol and testosterone inhibit quorum sensing (14). The influence of sex steroids on clinical disease is exemplified in the genetic condition cystic fibrosis (CF), where elevated 17β-estradiol correlates with the emergence of the deleterious mucoid phenotype of P. aeruginosa, a variant associated with poorer lung function and increased mortality, suggesting a key role for sex steroids on the bacterial pathogenicity (8). Despite these links, the mechanisms that underpin the specific roles of sex steroids on bacterial physiology and pathogenicity remain unknown. The presence of receptor proteins, such as LasR in P. aeruginosa, TraR in Escherichia coli, and even some ubiquitous efflux transporters such as AcrAB-TolC or ErmAB-TolC, that may recognize mammalian sex hormones has been suggested; however, no binding interactions or molecular mechanisms leading to virulent bacterial phenotypes are reported (5, 14–17).

Here, we provide evidence, using both *in vitro* and *in vivo* models, that natural and synthetic sex steroid hormones modulate P. aeruginosa disease-causing capabilities by inducing bacterial membrane stress responses and production of outer membrane vesicles (OMVs). The steroid concentration required to induce these bacterial responses inversely correlates with the hydrophobic nature of the compound. We propose that clinical isolates may be categorized as hormone-responsive or not depending on their membrane fluidic properties, which bears therapeutic potential in the treatment of P. aeruginosa chronic lung infections. This work offers fresh insight into our understanding of sex differences in the susceptibility and response to infections caused by P. aeruginosa in the clinic and highlights the potential impact of nonantimicrobial stressors on infectious disease outcomes.

## RESULTS

### Sex steroid hormones promote the virulence of P. aeruginosa.

**FIG 1:**
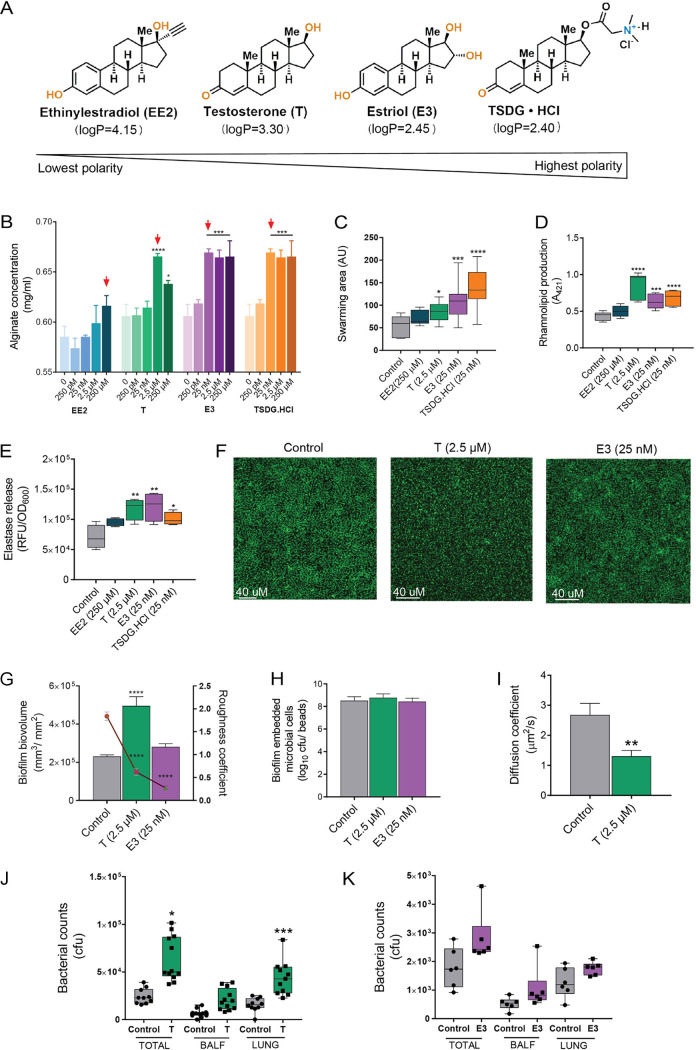
Sex steroids promote Pseudomonas aeruginosa virulence *in vitro* and reduce lung clearance *in vivo.* (A) Chemical structures of the various sex steroid hormones with associated logP values. (B) P. aeruginosa PAO1 was cultured for 16 to 24 h in tryptic soy broth (TSB) medium supplemented with ethanol (vehicle control) or the respective sex steroid hormones at concentrations varying from 250 pM to 250 μM, and alginate production was assessed. Sex steroids significantly promote P. aeruginosa *in vitro* production of alginates in a concentration-dependent manner with an inverse relationship with molecular polarity. (C to I) P. aeruginosa PAO1 was cultured for 16 to 24 h in TSB medium supplemented with ethanol (vehicle control) and the respective sex steroid hormones (at optimal concentrations varying from 25 nM to 250 μM). Changes in virulence were assessed by (C) increased swarming motility, (D) increased rhamnolipid production, (E) elevated elastase levels, and (F) altered biofilm architecture with (G) reduced roughness coefficient and increased biovolumes, (H) unchanged microbial cell burden, and (I) reduced diffusivity for the neutral molecule of dextran (150 kDa) in the matrix. Each experiment was performed in triplicate, and results are presented as the geometric mean and standard error (B, G to I) or box and whisker plots with median (horizontal bar), interquartile range (boxes), and minimum and maximum (whiskers) (C to E). Statistical significance was determined by one-way ANOVA corrected using Dunnett’s test for multiple comparisons. *, *P* < 0.05; **, *P* < 0.01; ***, *P* < 0.001; ****, *P* < 0.0001. (J and K) P. aeruginosa PAO1 was cultured for 24 h in Luria-Bertani medium supplemented with ethanol (vehicle control), testosterone (2.5 μM), or estriol (25 nM). Then, 10^6^ CFU were used to infect the lungs of BALB/c female mice (6 to 10 weeks old) intranasally. Bacterial cell burden measured at 6 h postinoculation in bronchoalveolar lavage fluid (BALF) revealed a significantly reduced P. aeruginosa lung clearance with (J) testosterone (2.5 μM) and a trend toward reduced clearance with (K) estriol (25 nM). Each experiment was performed in duplicate, and results are presented as box and whisker plots with median (horizontal bar), interquartile range (boxes), and minimum and maximum (whiskers). Statistical significance was determined by one-way ANOVA corrected using Tukey’s test for multiple comparisons. *, *P* < 0.05; ***, *P* < 0.001. EE2, ethinylestradiol; T, testosterone; E3, estriol; TSDG.HCl, 17β-N,N-dimethylglycinate testosterone hydrochloride; AU, arbitrary unit; RFU, relative fluorescence unit.

The female sex steroid hormone estrogen has been known to induce alginate production by P. aeruginosa, which is crucial for the mucoid conversion and survival of the bacteria in the human host (8, 14). Thus, we first assessed the ability of four different sex steroid hormones to alter P. aeruginosa alginate production. The tested compounds varied in polarity and included the synthetic hormone ethinylestradiol (EE2) (logP, 4.15) (18) (least polar), testosterone (T) (logP, 3.30) (19), the pregnancy-related estrogen estriol (E3) (logP, 2.45) (20), and a water-soluble synthetic and hydrophilic derivative of testosterone, TSDG.HCl (logP, 2.40) (21) (most polar) (Fig. 1A). Using a range of concentrations varying from 250 pM to 250 μM, we observed that while all compounds induced P. aeruginosa PAO1 alginate production, the concentration required to achieve the maximal response for each compound decreased as their molecular polarity increased (i.e., hydrophobicity decreased) (Fig. 1B). Thus, the most polar compounds, TSDG.HCl and estriol, required concentrations of ≈25 nM to significantly increase PAO1 alginate production, while molecules with lower polarity, testosterone and EE2, required 2.5 μM and 250 μM, respectively. Their effect was further confirmed by an increased swarming motility and rhamnolipid and elastase production, three major virulence factors produced by P. aeruginosa (Fig. 1C to E and Fig. S1A-C). The responses found were not attributable to differences in inocula or bacterial growth rates, as results were normalized at the time of cell harvest (see methods), and growth kinetics did not show any significant differences when P. aeruginosa was cultured with the different steroid compounds (Fig. S1D). Since it has been suggested that swarming motility has an impact on biofilm formation (22, 23), we next studied P. aeruginosa PAO1 biofilms grown in a hormone-rich environment. We observed a significantly reduced roughness coefficient of biofilms grown in the presence of testosterone (2.5 μM) or estriol (25 nM), illustrating a flat homogeneous architecture of the bacteria compared to vehicle control (ethanol) (Fig. 1F to G). Changes in the biofilm 3-dimensional morphology were not associated with differences in growth rates, as biofilms grown in the presence of sex steroids showed comparable numbers of viable biofilm embedded cells compared to ethanol (vehicle control) (Fig. 1H). However, biofilms grown in the presence of hormones had significantly increased biovolumes, a finding that is consistent with the increased alginate production found earlier (Fig. 1B and G). This shift in biofilm architecture further translated into an altered matrix and its related diffusion properties when tested with the neutral dextran molecule (150 kDa) that showed a significantly decreased molecular diffusion across PAO1 biofilms grown in the presence of testosterone compared to ethanol (vehicle control) (1.30 ± 0.97 μm^2^/s versus 2.68 ± 1.82 μm^2^/s, respectively; *P* < 0.05) (Fig. 1I).

10.1128/mBio.01774-20.2 FIG S1 Sex steroids modulate Pseudomonas aeruginosa virulence *in vitro* in a concentration-dependent manner, inversely related to their polarity and independent of the bacterial growth rate. (A to C) P. aeruginosa PAO1 was cultured for 16 to 24 h in tryptic soy broth (TSB) medium supplemented with ethanol (vehicle control) or the respective sex steroids: testosterone, estriol, 17β-N,N-dimethylglycinate testosterone hydrochloride (TSDG.HCl), or ethinylestradiol (EE2) at concentrations ranging from 250 pM to 250 μM, and virulence was assessed by (A) swarming motility, (B) rhamnolipid, and (C) elastase production. Compounds with higher polarity required lower concentrations to promote the production of virulence factors. (D) P. aeruginosa PAO1 was cultured for 18 h in TSB medium supplemented with ethanol (vehicle control) or the respective sex steroids: testosterone, estriol, TSDG.HCl, or EE2 at optimal concentrations that promote virulence. Optical density (OD_600_) measurements illustrate that sex steroid exposure does not alter the PAO1 growth rate. Each experiment was performed in triplicate, and results are presented as the geometric mean and standard error. Statistical significance was determined by one-way ANOVA corrected using Dunnett’s test for multiple comparisons with *P* < 0.05 (*), *P* < 0.01 (**), *P* < 0.001 (***), and *P* < 0.0001 (****). AU, arbitrary unit; RFU, relative fluorescence unit; T, testosterone; E3, estriol; EE2, ethinylestradiol; TSDG.HCl, 17β-N,N-dimethylglycinate testosterone hydrochloride. Download FIG S1, TIF file, 2.3 MB. Copyright © 2020 Vidaillac et al. 2020 Vidaillac et al. This content is distributed under the terms of the Creative Commons Attribution 4.0 International license.

To study the effects of steroid hormones *in vivo*, we used mouse models of lung infection where PAO1 bacteria were inoculated intranasally. By comparing PAO1 preexposed to ethanol (vehicle control) with treatment with the respective hormones (testosterone at 2.5 μM and estriol at 25 nM), we observed a significant increase in bacterial counts (CFU) in the lungs of mice treated with testosterone (*P* < 0.0001) and a trend toward significance with estriol (Fig. 1J and K). This occurred even though it was a short-term experiment (6 h) and P. aeruginosa is not a natural pathogen and typically results in variable infections. These data are consistent with our *in vitro* observations, suggesting an *in vivo* persistence due to sex steroid exposure. We did not detect any significant inflammatory changes in the bronchoalveolar lavage fluid over this time period (Fig. S2).

10.1128/mBio.01774-20.3 FIG S2 Preexposure to the sex steroids testosterone and estriol does not induce host immune responses to Pseudomonas aeruginosa *in vivo*. (A and B) P. aeruginosa PAO1 was cultured for 24 h in tryptic soy broth (TSB) medium supplemented with ethanol (vehicle control), testosterone (2.5 μM), or estriol (25 nM). Then, 10^6^ CFU were used to infect the lungs of BALB/c female mice (6 to 10 weeks old) intranasally. Host immune cells (leucocytes, macrophages, and neutrophils) obtained from bronchoalveolar lavage fluid (BALF) collected 6 h postinoculation illustrate no significant difference following microbial preexposure to (A) testosterone (2.5 μM) or (B) estriol (25 nM). Each experiment was performed in duplicate, and results are presented as box and whisker plots with median (horizontal bar), interquartile range (boxes), and minimum and maximum (whiskers). Statistical significance was determined by one-way ANOVA using uncorrected Fisher’s least significant difference (LSD) test. T, testosterone; E3, estriol. Download FIG S2, TIF file, 1.8 MB. Copyright © 2020 Vidaillac et al. 2020 Vidaillac et al. This content is distributed under the terms of the Creative Commons Attribution 4.0 International license.

### Sex steroids promote P. aeruginosa virulence through the *muc* operon.

**FIG 2:**
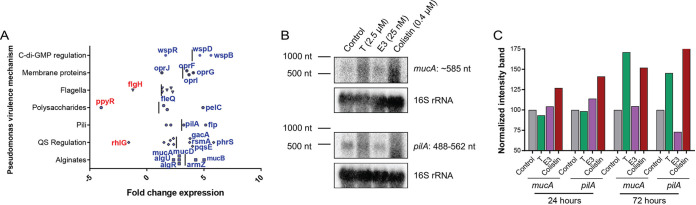
The sex steroids testosterone and estriol induce Pseudomonas aeruginosa virulence through molecular signaling pathways controlled by surface sensing and membrane stress. (A) P. aeruginosa PAO1 was cultured for 16 to 24 h in tryptic soy broth (TSB) medium supplemented with ethanol (vehicle control) or testosterone (2.5 μM) and subjected to RNA sequencing. Significant increases in key virulence genes (marked in blue) involved in surface sensing (c-di-GMP regulation, pili), membrane stress (polysaccharides, alginates, quorum sensing, membrane proteins), and motility (flagella) illustrate the global regulation driven by testosterone. Three genes involved in flagellar motility, rhamnolipids, and polysaccharide regulation were found to be significantly downregulated (marked in red). All the genes reported with a significant change in expression (defined as a >0.5-fold increase or decrease compared to the control) are represented. (B and C) P. aeruginosa PAO1 was cultured for 24 to 72 h in TSB medium supplemented with ethanol (vehicle control), testosterone (2.5 μM), estriol (25 nM), or colistin (0.4 μM, 1/4 MIC) as a positive control for the membrane stress response. Northern blot gels (B) show increased *mucA* and *pilA* mRNA expression following testosterone, estriol, and colistin exposure, further illustrated by their (C) corresponding normalized band intensities. T, testosterone; E3, estriol.

Since our data demonstrate that steroid treatment of P. aeruginosa affects its virulence mechanisms, we next used transcriptomic analyses of the bacteria to identify the potential molecular mechanism(s) that drive these effects. Exposure of PAO1 to testosterone (Fig. 2A) led to major transcriptomic alterations (measured as fold change [FC] expression > 1.5) in key virulence genes involved in surface sensing (c-di-GMP regulation and pili), membrane stress (polysaccharides, alginates, quorum sensing, and membrane proteins), and motility (flagella). These results suggest that global regulatory mechanisms that are activated by molecular signaling pathways are induced by P. aeruginosa envelope stress upon sex steroid exposure. Using colistin, a known membrane stress inducer, as a positive control (24), Northern blot analysis of PAO1 confirmed this (Fig. 2B and C). Thus, following testosterone (2.5 μM), estriol (25 nM), and colistin (0.4 μM) treatments, we observed increased expressions of *mucA*, a gene encoding an anti-sigma factor that controls alginate biosynthesis and mucoid conversion (25), and *pilA*, a gene encoding pilin, the main constituent of type IV pili, key for surface motility, cell adhesion, and biofilm formation (26).

**FIG 3:**
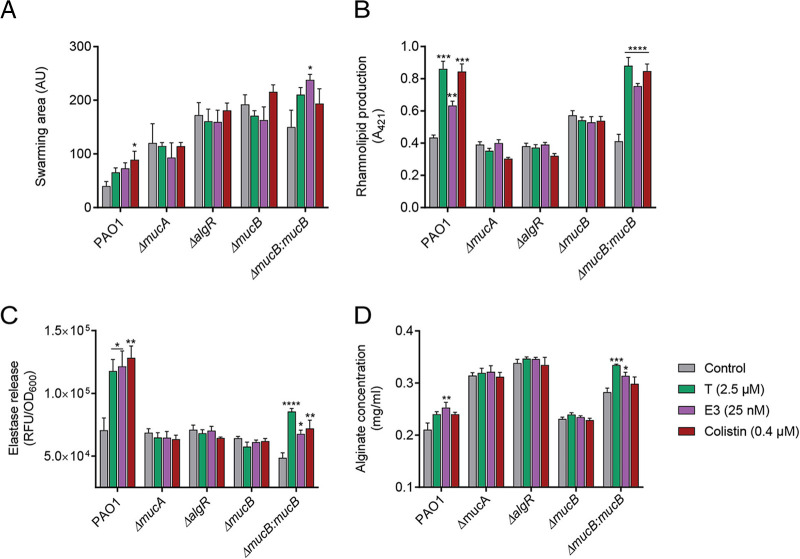
The sex steroid hormones testosterone and estriol induce enhanced Pseudomonas aeruginosa motility, rhamnolipid, elastase, and alginate production through the *muc* operon. (A) P. aeruginosa PAO1 and mutants lacking genes from the *muc* operon (*algR*, *mucA*, and *mucB*) were cultured for 24 h in tryptic soy broth medium supplemented with ethanol (vehicle control), testosterone (2.5 μM), estriol (25 nM), or colistin (0.4 μM, 1/4 MIC), and virulence was assessed. Testosterone and estriol induced increases in (A) swarming motility, (B) rhamnolipids, (C) elastase, and (D) alginate production in PAO1 but not in the *mucA*, *mucB*, or *algR* mutants, and the phenotype was restored following *mucB* complementation. Each experiment was performed in triplicate, and results are presented as the geometric mean and standard error. Statistical significance was determined by one-way ANOVA corrected using Dunnett’s test for multiple comparisons. *, *P* < 0.05; **, *P* < 0.01; ***, *P* < 0.001; ****, *P* < 0.0001. T, testosterone; E3, estriol; AU, arbitrary unit; RFU, relative fluorescence unit.

To identify proteins that bind to steroids, we next performed coimmunoprecipitation assays of testosterone and 17β-estradiol with P. aeruginosa lysates combined with mass spectrometry (MS) analysis. This revealed 11 and >50 protein hits for testosterone and 17β-estradiol, respectively (Table S1A). The P. aeruginosa master regulator of virulence, Vfr, was identified as a testosterone-associated protein and therefore subjected to further analysis. In addition to assessing the impact of steroid exposure on P. aeruginosa lacking *vfr* (Fig. S3), further evaluation of protein-ligand interactions was performed using surface plasmon resonance (SPR). No direct interaction between Vfr and the main steroid hormones was found, suggesting that the sex hormones do not act directly on Vfr, but rather, act through a molecular pathway upstream of the *vfr* regulatory network (Fig. S4). Further analyses of the MS results identified *mucB* as a potential candidate. The *mucABCD* operon has been shown to influence P. aeruginosa virulence, mucoid conversion, and host persistence during chronic infections (27) (Table S1A). Analysis of *mucB* and steroid binding using SPR revealed no direct interaction (Fig. S4). We thus explored if the observed effects of steroids on P. aeruginosa virulence may be due to disruption of *mucB*-related signaling pathways. We exposed PAO1, its *mucB* mutant knockout derivative, and a *mucB* complemented strain to ethanol (vehicle control), testosterone (2.5 μM), estriol (25 nM), and colistin (0.4 μM) and assessed swarming motility, rhamnolipids, elastase, and alginate production (Fig. 3A to D). Interestingly, in the absence of *mucB*, PAO1 did not respond to testosterone, an effect that was restored by *mucB* complementation. To further confirm this hypothesis, we then investigated the role of other related genes in the *muc* operon and alginate biosynthetic pathways, *mucA* and *algR* (Fig. 3A to D). Using a PAO1 mutant knockout in *mucA* or *algR*, we observed that both steroids and colistin induced significant increases in swarming motility, rhamnolipids, elastase, and alginate production in wild-type (WT) PAO1 but not in the mutants, confirming that the effects of sex steroid exposure observed on P. aeruginosa occur through the *muc* operon.

10.1128/mBio.01774-20.4 FIG S3 The sex steroid hormones testosterone and estriol do not induce Pseudomonas aeruginosa motility or rhamnolipid, elastase, and alginate production through the master regulator for virulence *vfr*. (A to D) P. aeruginosa PAO1, its mutant lacking *vfr*, and the *vfr* complemented strain were cultured for 24 h in tryptic soy broth (TSB) medium supplemented with ethanol (vehicle control), testosterone (2.5 μM), estriol (25 nM), or colistin (0.4 μM), and virulence was assessed. Testosterone and estriol induce increased (A) swarming motility, (B) rhamnolipid, (C) elastase, and (D) alginate production in the PAO1, *vfr-*mutant, and *vfr* complemented strains. Each experiment was performed in triplicate, and results are presented as the geometric mean and standard error. Statistical significance was determined by one-way ANOVA corrected using Dunnett’s test for multiple comparisons. *, *P* < 0.05; **, *P* < 0.01; ***, *P* < 0.001; ****, *P* < 0.0001. T, testosterone; E3, estriol; AU, arbitrary unit; RFU, relative fluorescence unit. Download FIG S3, TIF file, 2.9 MB. Copyright © 2020 Vidaillac et al. 2020 Vidaillac et al. This content is distributed under the terms of the Creative Commons Attribution 4.0 International license.

10.1128/mBio.01774-20.5 FIG S4 The sex steroid hormones testosterone and estriol do not interact with Vfr or MucB. (A to C) Pseudomonas aeruginosa Vfr protein was purified and subjected to cAMP (control), testosterone (T), and estriol (E3) binding using surface plasma resonance (SPR). Under our experimental conditions, the dissociation constant (Kd) was estimated at (A) 6.2 to 11 μM for cAMP, (B) 1.7 to 1.8 mM for T, and (C) 750 to 782 μM for E3. (D to F) Pseudomonas aeruginosa MucB protein was purified and subjected to MucA (control), testosterone (T), and estriol (E3) binding using surface plasma resonance (SPR). (D) Under our experimental conditions, the dissociation constant (Kd) of MucA was estimated at 2.93 μM, while no interaction was observed between (E) T and (F) E3 at concentrations ranging from 0.06 nM to 10 μM. Download FIG S4, TIF file, 2.9 MB. Copyright © 2020 Vidaillac et al. 2020 Vidaillac et al. This content is distributed under the terms of the Creative Commons Attribution 4.0 International license.

10.1128/mBio.01774-20.9 TABLE S1 (A) Coimmunoprecipitation *of* Pseudomonas aeruginosa with testosterone or estradiol and their identified protein binders. P. aeruginosa PAO1 was cultured for 24 h in tryptic soy broth medium supplemented with ethanol (vehicle control), testosterone (2.5 μM), or estradiol (25 nM). Membrane extracts were subjected to coimmunoprecipitation and mass spectrometry (MS), and results were compared to the control (ethanol). Experiments were run in duplicate, and proteins present in duplicate experiments are reported. MS analysis revealed 11 protein hits for testosterone and >50 protein hits for estradiol. (B) The sex steroid hormones estriol and testosterone alter outer membrane vesicle (OMV) protein content and released amounts by Pseudomonas aeruginosa PAO1. P. aeruginosa PAO1 was cultured for 24 h in tryptic soy broth medium supplemented with ethanol (vehicle control), testosterone (2.5 μM), or estriol (25 nM). OMVs were extracted from the resulting supernatant and further purified as described in Materials and Methods. OMV extracts were loaded into a sodium dodecyl sulfate PAGE gel and subjected to proteomic analysis with a threshold of 30 for coverage. T, testosterone; E3, estriol; OM, outer membrane; EC, extracellular; P, periplasm; C, cytoplasm; U, Unknown. Download Table S1, DOCX file, 0.03 MB. Copyright © 2020 Vidaillac et al. 2020 Vidaillac et al. This content is distributed under the terms of the Creative Commons Attribution 4.0 International license.

### Sex steroids induce P. aeruginosa membrane stress and production of outer membrane vesicles.

**FIG 4:**
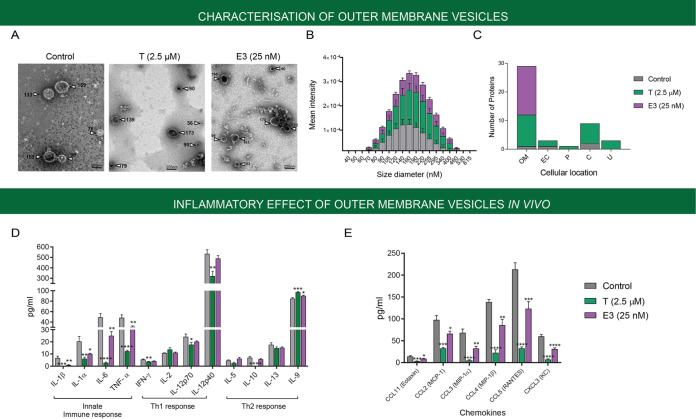
The sex steroid hormones testosterone and estriol induce release of Pseudomonas aeruginosa outer membrane vesicles with diminished inflammatory capability. P. aeruginosa PAO1 was cultured for 24 h in Tryptic soy broth medium supplemented with ethanol (vehicle control), testosterone (2.5 μM), or estriol (25 nM). Outer membrane vesicles (OMVs) were extracted from the resulting supernatant and further purified as described in Materials and Methods. (A) Representative electron photomicrographs of extracted OMVs from cultures exposed to ethanol (vehicle control), testosterone (2.5 μM), or estriol (25 nM) (arrows indicate OMVs with their respective vesicle size in nm). (B) OMV amount, but not size, differs under sex steroid stress (range, 80 to 400 nm). (C) OMV extracts subjected to proteomic analysis (Table S2B) reveal significant differences in protein composition and diversity and consist of predominantly outer membrane (OM) proteins. (D and E) Extracted OMVs (10 ng total protein) were administered to the lungs of female C57BL/6 mice (*n* = 5, 6 to 8 weeks old) using the intratracheal route. After 24 h, bronchoalveolar lavage fluid was obtained, and inflammatory (D) cytokines and (E) chemokines were measured, which illustrate a significantly diminished inflammatory response from OMVs secreted under sex steroid stress. Each experiment was performed in triplicate, and results are presented as the geometric mean and standard error. Statistical significance was determined by one-way ANOVA corrected using Dunnett’s test for multiple comparisons. *, *P* < 0.05; **, *P* < 0.01; ***, *P* < 0.001; ****, *P* < 0.0001. T, testosterone; E3, estriol; OM, outer membrane; EC, extracellular; P, periplasm; C, cytoplasm; U, unknown.

P. aeruginosa, like many other Gram-negative bacteria, secretes OMVs in response to environmental stresses (28–30). We therefore evaluated the impact of sex steroid exposure (estriol and testosterone) on OMV production. We found that P. aeruginosa PAO1 produces significantly more OMVs (2 to 3 times as many) in response to steroids (at their optimal concentrations) compared to the control (ethanol) (Fig. 4A and B). The size distribution of the secreted vesicles (80 to 400 nm) remained unchanged, but there were major differences in their protein composition. Proteomic analysis of purified OMV extracts exposed to sex steroids revealed a diverse array of predominantly outer membrane proteins, with significant alterations of the outer membrane lipid/protein composition and changes to cell envelope fitness compared to the control (Fig. 4C and Table S1B). While some proteins, including OmpA and flagellin, were detected in the OMVs produced under both control (ethanol) conditions and treatment with steroids, lipoproteins (PA2853, PA4423), efflux transporters, and type 6 secretion systems (T6SS) were more prominent after hormone exposure, suggesting that steroids induce alterations of the bacterial envelope (Table S1B). Interestingly, OMVs secreted during hormonal stress carried an uncharacterized protein, PA2462. This protein, previously identified in P. aeruginosa OMVs (31), also shares high sequence homologies with a filamentous hemagglutinin protein described in Bordetella pertussis that has been associated with bacterial adherence to ciliated eukaryotic cells (32). Next, we evaluated the *in vivo* effects of these OMVs by instilling purified extracts intratracheally in a model using C57BL/6 female mice (6 to 8 weeks old). Lung tissue and bronchoalveolar lavage fluid (BALF) harvested at 24 h postinoculation illustrated the inflammatory response triggered by P. aeruginosa OMVs (Fig. 4D and E). Challenge with OMVs isolated during hormone treatment induced significantly lower cytokine (IL-1α, IL-1β, IL-6, and TNF-α) and chemokine (CCL2-5 and CXCL3) responses in the BALF 24 h postinfection than the control (*P* < 0.001). Lung histology correlated with these observations, and the pulmonary damage was most prominent after challenge with estriol-derived OMVs (Fig. S5). Taken together, our *in vitro* and *in vivo* data provide strong evidence that sex steroid hormones affect the disease-causing capability of P. aeruginosa.

10.1128/mBio.01774-20.6 FIG S5 Sex steroid hormones suppress the neutrophilic host defense response toward bacterial infection in mouse models administered with outer membrane vesicles (OMVs) produced from hormone-exposed Pseudomonas aeruginosa. Mice were administered with intratracheally delivered OMVs isolated from P. aeruginosa PAO1 treated with the indicated sex steroid hormones. Bronchoalveolar lavage fluid (BALF) and lung tissue were harvested 24 h post-OMV inhalation. (A) OMVs from testosterone (T) exposure showed a marked decrease in neutrophil/macrophage ratio compared to the estriol (E3) and control groups. (B) Lung tissue sections from the indicated groups illustrate a thickening of the bronchiolar epithelium (red arrow) and alveolar inflammation (black arrow) in the control compared to the estriol and testosterone groups. Download FIG S5, TIF file, 2.2 MB. Copyright © 2020 Vidaillac et al. 2020 Vidaillac et al. This content is distributed under the terms of the Creative Commons Attribution 4.0 International license.

10.1128/mBio.01774-20.10 TABLE S2 (A) Summary of clinical Pseudomonas aeruginosa isolates used in this study. (B) Summary of Pseudomonas aeruginosa strains, plasmids, and primers used in this study. Download Table S2, DOCX file, 0.02 MB. Copyright © 2020 Vidaillac et al. 2020 Vidaillac et al. This content is distributed under the terms of the Creative Commons Attribution 4.0 International license.

### Variation in membrane fluidity among P. aeruginosa clinical isolates explains sex-hormone responsiveness.

**FIG 5:**
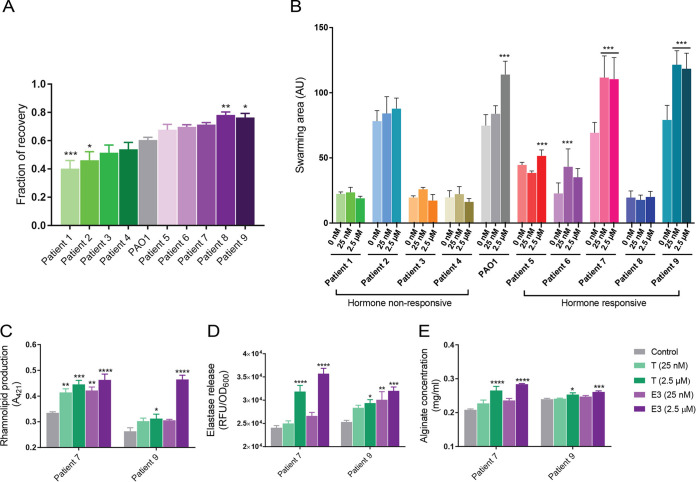
Variations in membrane fluidity among Pseudomonas aeruginosa clinical isolates explains sex hormone responsiveness. (A and B) Membrane fluidity (assessed using fluorescence recovery after photobleaching [FRAP]) of nine clinical P. aeruginosa isolates from nine patients was compared to PAO1. Four of the nine isolates (P. aeruginosa from patients 1 to 4) demonstrate lower membrane fluidity, while the other five (P. aeruginosa from patients 5 to 9) show higher membrane fluidity compared to PAO1. (B) Clinical P. aeruginosa isolates were cultured for 24 h in tryptic soy broth (TSB) medium supplemented with ethanol (vehicle control) or testosterone at two concentrations (physiological concentration, 25 nM; supraphysiological but optimal concentration for PAO1, 2.5 μM). The motility response (swarming) was assessed as an indicator of the sex steroid virulence response. The four isolates with the lowest membrane fluidity compared to PAO1 (P. aeruginosa from patients 1 to 4) did not demonstrate any motility changes following testosterone exposure (termed “nonresponsive”). In contrast, four of the five isolates (P. aeruginosa from patients 5 to 7 and patient 9) demonstrated significantly increased motility (and higher membrane fluidity) compared to PAO1 following testosterone exposure (termed “hormone-responsive”). (C to E) Two hormone-responsive clinical isolates (patients 7 and 9) were selected and assessed for further virulence studies. Strains were cultured for 24 h in TSB medium supplemented with ethanol (vehicle control), testosterone, or estriol, each at two separate concentrations (25 nM and 2.5 μM as described above). Both hormone-responsive strains exhibited significant upregulation of virulence markers upon hormone exposure as evidenced by elevated (C) rhamnolipids, (D) elastase, and (E) alginate production. Each experiment was performed in triplicate, and results are presented as the geometric mean and standard error. Statistical significance was determined by one-way ANOVA corrected using Dunnett’s test for multiple comparisons. *, *P* < 0.05; **, *P* < 0.01; *** *P* < 0.001; ****, *P* < 0.0001. T, testosterone; E3, estriol; AU, arbitrary unit; RFU, relative fluorescence unit.

To study the clinical relevance of our findings and determine if the effect of sex steroid hormones on P. aeruginosa virulence differs between clinical strains due to differences in their membrane fluidity, we performed fluorescence recovery after photobleaching (FRAP) studies. This technique is widely used to measure the mobility and interaction of fluorescently labeled biological molecules within living membranes. As a first measurement, we used the WT PAO1 strain, as well as the *mucB* mutant and its complemented derivative. Since the *muc* operon plays a central role in the response to membrane stress and production of OMVs (33), we speculated that mutations in this operon will translate into significant alteration of membrane fluidity. Consistent with this hypothesis, we found that the *mucB* mutant had a significant reduction in the fraction of recovery compared to PAO1, which was restored to wild-type levels in the *mucB* complemented strain (Fig. S6). Next, we assessed the recovery fractions of nine clinical isolates of P. aeruginosa isolated from different patients (Table S2A). Four of the nine isolates (44%) exhibited lower fractions of recovery compared to WT PAO1, two of which were statistically significant (patient isolates 1 and 2) (Fig. 5A). The five remaining isolates exhibited a higher fraction of recovery than WT PAO1 (patient isolates 5 to 9), two of which were statistically significant (patient isolates 8 and 9) (Fig. 5A). To test whether there was a correlation between the fraction of recovery (membrane fluidity) and steroid “susceptibility” of the isolates, we next assessed the impact of adding testosterone (at 25 nM and 2.5 μM) on their swarming motility (Fig. 5B). As expected, we found that the isolates with a higher fraction of recovery, except isolate 8, showed a significant increased swarming capacity, while those isolates with a lower fraction of recovery were nonresponsive to steroid treatment. To further confirm the FRAP findings, we assessed two clinical isolates (5 and 9) against a larger panel of P. aeruginosa virulence factors. We found that both strains had enhanced rhamnolipids, elastase, and alginate production in the presence of testosterone or estriol at concentrations ranging from 25 nM (physiological) to 2.5 μM (supraphysiological) (Fig. 5C to E). These data support that clinical isolates of P. aeruginosa respond differently to sex hormones depending on their membrane properties, which affects virulence.

10.1128/mBio.01774-20.7 FIG S6 The *muc* operon is an important determinant of Pseudomonas aeruginosa membrane fluidity. P. aeruginosa PAO1, a *mucB*-mutant, and a *mucB* complemented strain were cultured for 24 h in tryptic soy broth medium, and membrane fluidity was assessed by fluorescence recovery after photobleaching. The *mucB*-mutant P. aeruginosa illustrates a significantly decreased membrane fluidity compared to PAO1 or complemented *mucB* P. aeruginosa. Each experiment was performed in triplicate, and results are presented as the geometric mean and standard error. Statistical significance was determined by one-way ANOVA corrected using Dunnett’s test for multiple comparisons. **, *P* < 0.01. Download FIG S6, TIF file, 1.7 MB. Copyright © 2020 Vidaillac et al. 2020 Vidaillac et al. This content is distributed under the terms of the Creative Commons Attribution 4.0 International license.

## DISCUSSION

The present study provides molecular and cellular evidence that sex steroid hormones modulate P. aeruginosa virulence through membrane stress and release of OMVs, which depends on bacterial membrane properties. In agreement with published data, we illustrate that estrogens induce alginate production (8, 14). However, we also show that a range of sex steroids, including male, female, natural, and synthetic hormones, have the potential to modulate P. aeruginosa pathogenicity by promoting surface motility, rhamnolipids, elastase, and alginate production and formation of structurally different biofilms with significantly reduced permeability (14, 22, 34, 35). The effects of the steroids correlate with their molecular polarity, a physicochemical property dictating their ability to diffuse across bacterial membranes (36–38). With higher molecular polarity, the diffusivity becomes slower through the bacterial membrane because of increased interactions with proteins, lipids, and polysaccharides, which in turn, increases the membrane stress. Also, the required steroid concentration to induce membrane stress is lower with higher polarity. Thus, the polarity of the substance affects the P. aeruginosa membrane composition and integrity (39). Indeed, the most polar of the natural steroids, the pregnancy-related estrogen estriol required only nanomolar concentrations to alter P. aeruginosa virulence. These concentrations are physiological and have been previously demonstrated in bronchoalveolar lavage fluid (8). Although estriol is the predominant estrogen in pregnancy, it may also be detected in the lung following metabolism of estradiol by aromatase enzymes present in the airway epithelium (8, 40). In contrast, the least polar of all tested steroids, the synthetic estrogen used in oral contraception, ethinylestradiol, necessitated concentrations 1,000-fold higher than estriol to induce similar effects.

Furthermore, we found that the steroid-induced P. aeruginosa membrane change activates a global stress response controlled by the *muc* operon. This operon controls genes involved in *Pseudomonas* virulence such as surface motility (flagella function), biofilm formation, regulation of quorum sensing (*rhl*, *pqs*, and *las*), alginate biosynthesis, and type IV pili (41). Activation of quorum sensing contributes to shaping the bacterial community, including a switch to biofilms and inter- and intraspecies communication through the diffusion of autoinducer (AI) molecules (*pqs* and *las*). Increased production of AIs and alginate alters the outer membrane properties and promotes lipid raft formation and OMV release (33). Indeed, we observed increased production of OMVs in response to steroids. OMVs affect bacterial persistence and host immune responses and tissue damage (42). OMV release following sex steroid exposure represents an underappreciated and important bacterial virulence mechanism (28, 43). With a central role in cell communication, OMVs are key to biofilm formation and facilitating host-pathogen interactions, which is attributed to their proinflammatory properties (44–46). A defensive role has also been proposed, where OMVs inhibit the efficacy of antimicrobial agents that target the bacterial cell wall (24). In addition to release, the amount and composition of the OMVs were found to be altered and include proteins involved in bacterial virulence such as lipoproteins, toxins, and adhesins. Changes in OMV protein composition likely explain the altered inflammatory properties observed in the *in vivo* mouse models that support bacterial persistence in sex steroid-rich environments.

**FIG 6:**
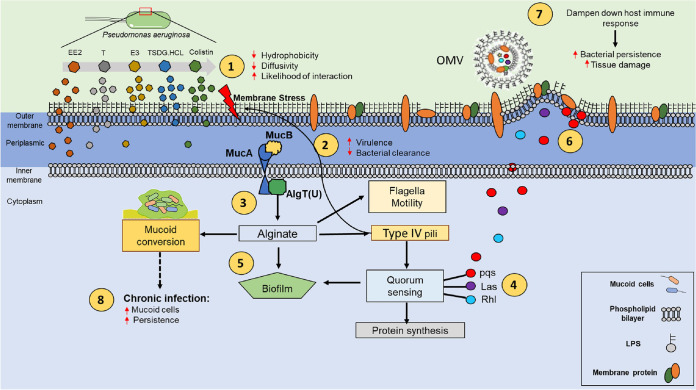
Proposed mechanistic model for sex steroid-driven regulation of Pseudomonas aeruginosa virulence and persistence. (1) The polarity of the various sex steroid hormones influences their ability to diffuse across the P. aeruginosa (lipid bilayer) membrane with their degree of impact on membrane dynamics dependent on the diffusion rate. More polar molecules such as estriol (with lower hydrophobicity) diffuse more slowly and therefore are more likely to interact with lipopolysaccharides and proteins in the bacterial cell envelope compared to less polar molecules such as testosterone or ethinylestradiol (with higher hydrophobicity), which diffuse more rapidly through the bacterial membrane. (2) Interactions between polar sex steroids, membrane proteins, and lipopolysaccharides are sensed by surface signaling systems, including the *muc* operon (*muc*A/B), leading to (3) activation of the alginate biosynthetic pathway through the *muc* operon, (4) upregulation of downstream genes involved in surface sensing (pili, c-di-GMP) and quorum sensing (*pqs*, *las*, *rhl*, *vfr*), and (5) biofilm formation (polysaccharides, alginates). (6) The increased secretion of quorum sensing autoinducer (AI) molecules coupled to increased membrane protein production alters membrane composition and fluidity and promotes “lipid raft” formation and membrane stress-inducing (7) OMV release that dampens host inflammatory responses and further promotes bacterial persistence and tissue damage. (8) In chronic P. aeruginosa infection with “hormone-responsive” strains, the constant pressure exerted by sex steroids, particularly on the bacterial envelope, promotes adaptive change, which aims to protect the bacterium by creating defensive barriers such as alginates that surround bacterial cells, leading to mucoid conversion with bacterial persistence and poorer clinical outcomes. EE2, ethinylestradiol; T, testosterone; E3, estriol; TSDG.HCl, 17β-N,N-dimethylglycinate testosterone hydrochloride; OMV, outer membrane vesicle; LPS, lipopolysaccharides.

Thus, our results support a model whereby sex steroids act as extracellular chemical signals received by the surface sensing regulatory network of P. aeruginosa (41) (Fig. 6) involving the *algT*-*muc* cluster, which is central to the control of bacterial virulence and responses to environmental stresses (47). Potentially, exposure to steroids *in vivo* over a long period may induce mutations in the *muc* operon that may reduce the fitness costs provoked by the steroid stress. This might lead to a selection of mucoid and persistent P. aeruginosa mutant strains that confer poorer clinical outcomes (8, 48). Further investigations are warranted to better understand the impact of sex steroid-induced OMVs on the course of P. aeruginosa infection, especially in the setting of a chronic inflammatory milieu.

Our findings here show clinical relevance since we observe a correlation between the membrane fluid dynamics of individual P. aeruginosa clinical isolates and their ability to respond to sex steroids. We show that the concentration and chemical nature of the steroid hormone shape the level of response together with the membrane properties of the exposed P. aeruginosa strain. This finding might explain sex differences observed across a range of lung diseases affected by chronic *Pseudomonas* infections (1, 2, 4). Females with CF have earlier colonization (8, 49–51) and mucoid conversion of P. aeruginosa (8, 52), which in turn, translates to poorer clinical outcomes and higher mortality (8, 52–54). However, this is not uniformly observed across individual CF females. Our work highlights that colonization with “hormone-responsive” strains may explain this phenomenon and, as such, relate to other chronic respiratory diseases complicated by P. aeruginosa infection. This work also furthers our understanding of the variable treatment responses observed between individual female patients affected by P. aeruginosa. Exploring whether a colonizing P. aeruginosa strain is responsive to hormonal stress may allow a more focused and tailored therapeutic approach based on sex steroids and gender, particularly in the era of antimicrobial resistance (55, 56). A further interesting aspect of our work is improved mechanistic insight into why CF females taking oral contraceptive pills experience fewer exacerbations and need for antibiotics (8, 57, 58). In these women, synthetic exogenous ethinylestradiol ([EE2] logP, 4.15) suppresses the more polar and endogenous estradiol ([E2] logP, 2.70) (59), which based on our data, would have less effect on inducing P. aeruginosa virulence and potentially translate to better patient outcomes.

Importantly, the observed effects on P. aeruginosa virulence are not specific to steroids. Colistin, an antimicrobial used in the treatment of multidrug-resistant P. aeruginosa infection, illustrated a comparable ability to that observed with sex steroids in modulating P. aeruginosa virulence. In agreement with previous studies, our data highlight the potential for colistin to exhibit deleterious effects on bacterial virulence if used at suboptimal concentrations against the targeted pathogen (60). Chemical, physiological, or therapeutic exposures including steroids and nonsteroidal or antimicrobial agents that may influence bacterial virulence and the course of infection should be considered when evaluating an optimal treatment strategy for any individual patient (61). Despite emerging evidence of potential sex bias in infection, therapeutic strategies and antimicrobial treatments remain unchanged and do not currently account for gender (1, 4, 5). Antimicrobial dosing is currently largely determined using *in vitro* and *in vivo* pharmacotherapeutic studies with environmental conditions focused on host immunity, the pathogen’s antimicrobial susceptibility, and the antimicrobial agents’ pharmacokinetic properties (62). However, patients who are treated with concomitant and sometimes combination therapies are at the same time exposed to their natural circulating hormones that, based on the data presented, confer an additional and largely unrecognized effect on pathogen virulence and evolution and antimicrobial susceptibility. Further investigations are therefore needed to better understand the impact of sex steroid hormones and other concomitant therapies (using polar molecules) that can induce a P. aeruginosa membrane stress response. These effects at an individual level may influence optimal antimicrobial dosing regimens and could become part of precision-medicine approaches for the treatment of infectious disease.

Gender differences are also reported with other clinically relevant Gram-negative pathogens (2, 63, 64), and existing work supports the findings presented in this study with P. aeruginosa (65–67). Higher bacterial survival was reported in ovariectomized rabbits infected with E. coli following treatment with estradiol and progesterone (65). Similarly, female virgin mice treated with estrogen or progesterone for 3 days illustrate significant increases in Salmonella enterica serovar Typhimurium survival (67). Although such work was not designed to assess the direct effects of sex steroids on bacterial pathogenicity, it is of interest that an envelope stress response mechanism does exist in both E. coli and *Salmonella* spp. to maintain membrane integrity and cell homeostasis and facilitate chronic colonization (68, 69). These mechanisms are controlled by the extracytoplasmic sigma factor rpoE or σE, which shares 66% and 91% homology, respectively, with P. aeruginosa AlgU (68).

Although this study comprehensively assesses the *in vitro* and *in vivo* effects of sex steroid hormones on P. aeruginosa virulence, there are further aspects that warrant future investigation. We did not investigate metabolomic changes that occur under sex steroid stress. P. aeruginosa secretes a range of signaling molecules to communicate with its surroundings and adapt its behavior to its environment. Such molecules, termed “auto-inducers” are secreted through their membrane, transported by OMVs, and affect membrane dynamics and integrity, in turn playing a central role in the host-pathogen relationship (70, 71). Therefore, studies focusing on metabolomic alterations in the bacterial membrane, and within OMVs, should be considered in future research including the production of quorum sensing (QS) molecules. The biofilm models presented in this study may not optimally reflect the *in vivo* lung state. Air-liquid interface cell cultures may be a model for future work that more accurately mimics the *in vivo* state (72). Finally, additional *in vivo* animal models more representative of a chronic infection state, while more complex to perform, may be valuable in future work to better understand how sex steroids influence P. aeruginosa invasion, colonization, and adaptation in the lungs over the longer term.

Our work unlocks novel academic and translational research avenues for the microbial endocrinology field, and our findings should be carefully considered in individuals receiving concomitant high-polarity therapeutics. The influence of sex steroids on membrane stress, bacterial virulence, and antimicrobial susceptibility demonstrated here for P. aeruginosa should also be considered for other clinically relevant Gram-negative pathogens.

## MATERIALS AND METHODS

### Ethics statement.

Approval for investigation of the isolates of P. aeruginosa, with linked pseudonymized patient metadata was given by the St. James’s/Tallaght University Hospital Ethics Committee (REC: 2017-01 list 1 [8]). Ethics approval for experiments conducted with BALB/c female mice was obtained from the University of Newcastle Animal Care and Ethics Committee (A-2012-233), and for those with C57BL/6 female mice, was obtained from the IACUC Committee of the Shenzhen Institute of Advanced Technology, Chinese Academy of Sciences (SIAT-IACUC-181225-YYS-LL-A0551).

### Bacterial strains and growth conditions.

The strains of P. aeruginosa used in this study are listed in Table S2. The strains included *algR* and *mucA* mutant derivatives obtained from the P. aeruginosa PAO1 transposon mutant two-allele library (University of Washington, Seattle, WA; Manoil laboratory), which was established with NIH funds (grant P30 DK089507) (73). A *vfr* deletion mutant was generated using a previously reported knockout system (74), and *vfr* and *mucB* complemented isolates were constructed by electroporation of pNIC *vfr* or pNIC *mucB* plasmids (Bio Basic, Inc.). Mutant selection was performed on gentamicin-containing agar plates (30 μg/ml). Bacterial strains were cultured in tryptic soy broth (TSB; Becton, Dickinson) at 37°C under aeration (200 rpm) unless stated otherwise. Medium was prepared in glass bottles previously washed with a solution of hydrochloric acid and rinsed at least twice with distilled water (dH_2_O) to remove any excess detergents or ions that may affect experimental reproducibility. Stock solutions of steroid hormones (Sigma-Aldrich) were prepared in ethanol (Thermo Fisher Scientific) and stored at −20°C, and new stocks were generated every 7 days. TSDG.HCl was synthesized as described in Supplemental Methods and Fig. S7. Colistin (Sigma-Aldrich) was prepared fresh before each experiment, solubilized in sterile dH_2_O, and further diluted to the desired concentrations in 100% molecular-grade ethanol (Thermo Fisher Scientific) for experimental consistency.

10.1128/mBio.01774-20.8 FIG S7 Synthesis of the testosterone water-soluble derivative, testosterone 17β-N,N-dimethylglycinate hydrochloride salt (TSDG.HCl). (A) Scheme summarizing the different steps of synthesis of the testosterone water-soluble derivative, TSDG.HCl. (B to D) Nuclear magnetic resonance spectroscopy analysis of testosterone (T), TSDG, and its water-soluble salt TSDG.HCl. Download FIG S7, TIF file, 2.3 MB. Copyright © 2020 Vidaillac et al. 2020 Vidaillac et al. This content is distributed under the terms of the Creative Commons Attribution 4.0 International license.

### P. aeruginosa surface motility assay.

The swarming motility of P. aeruginosa strains was assessed using the previously described methodology with some modifications (75). Swarming medium was prepared by mixing 0.5 % (wt/vol) Bacto agar (Axil Scientific, 1st Base), 0.5 % (wt/vol) peptone (Becton, Dickinson), and 0.2 % (wt/vol) yeast extract (Axil Scientific, 1st Base). Following agar sterilization, the medium was supplemented with 1.0 % (vol/vol) glucose (Axil Scientific, 1st Base) and left to cool at 45°C in a water bath. Molten agar (20 ml) was poured into 15-cm petri dishes and dried open for 5 min in a fumigated hood. Overnight cultures (2 ml) of P. aeruginosa grown in the presence of ethanol (control), steroids, or colistin were centrifuged (8,000 rpm, 10 min). Pellets were spot inoculated at the center of the agar surface using a sterile toothpick, and plates were then incubated for 16 h at 37°C. Images of swarming colonies were taken using a Gel Doc XR + system (Bio-Rad), and the extent of swarming was determined by measuring the swarming area using ImageJ software (76). At least, three biological and two technical replicates were performed to ensure the reproducibility of each experiment.

### Extraction and quantification of P. aeruginosa virulence factors.

P. aeruginosa strains were cultured in TSB at 37°C under aeration (200 rpm) in the presence of ethanol (control), steroids, or colistin. After measuring cell density (optical density at 600 nm [OD_600_]), overnight cultures (10 ml) were centrifuged (10,000 rpm, 10 min). Supernatants were collected and filter sterilized through 0.2-μm filters (Pall Life Sciences) and used for rhamnolipids, elastase, and alginate extraction and quantification.

### Rhamnolipids.

Rhamnolipids were extracted and further quantified using a previously reported methodology with modifications (77). Briefly, rhamnolipids were extracted from supernatants (8 ml) using diethyl-ether. Extraction was performed twice, and the collected organic fractions were left to evaporate overnight. The resulting white solid precipitates were dissolved in water (100 μl) and mixed with a 0.19% (wt/vol) orcinol solution (900 μl) freshly prepared in 50% H_2_SO_4_. Samples were heated at 80°C for 10 min and cooled to room temperature for 10 min before the absorbance at 421 nm was measured. The results were normalized using the OD_600_ of the overnight cultures. Three biological and three technical replicates were performed to ensure experimental reproducibility.

### Elastase.

The elastase present in the filtered supernatants (1 ml) was measured using an elastase assay kit (EnzChek) according to the manufacturer’s instructions. TSB medium was used as the negative control, while elastase provided in the kit was the positive control. Fluorescence from each reaction (expressed in relative fluorescence units [RFU]) was measured using a microplate reader (Tecan Infinite 2000) and normalized using the OD_600_ of the overnight cultures. Three biological and three technical replicates were performed to ensure experimental reproducibility.

### Alginates.

The alginates present in the filtered supernatants were determined using the uronic acid/carbazole reaction as previously described (8). Supernatants (1 ml) were mixed with 2% cetylpyridinium chloride (500 ml) to precipitate alginates. Alginates were then collected by centrifugation (10,000 × *g* for 10 min at room temperature) and resuspended in 500 μl of cold (–20°C) isopropanol for 1 h. Repeat centrifugation was performed (10,000 × *g* at 4°C for 10 min), and the alginate pellets were resuspended in 1 M NaCl (500 μl) for alginate quantification. A solution of purified alginate (50 μl) was mixed with 200 μl of sodium tetraborate/sulfuric acid reagent (25 mM in 2 M H_3_BO_3_ in sulfuric acid), and the mixture was heated to 100°C for 10 min. Following cooling for 15 min, 50 μl 0.125% carbazole reagent (in 100% ethanol) was then added, and the mixture was heated to 100°C for another 10 min. Following cooling for 15 min, alginate concentrations were determined spectrophotometrically at 550 nm using a standard curve with alginic acid. Three biological and two technical replicates were performed to ensure experimental reproducibility.

### Biofilm culture and imaging.

Colonies of P. aeruginosa were suspended in fresh sterile TSB (5 ml), and inoculum (200 μl) was distributed across 8-well Ibidi μ-slides (Martinsried, Germany). Individual samples were supplemented with either 1 μl molecular-grade ethanol (control), steroids, or colistin at the desired concentrations. Cultures were placed at 37°C under static conditions for biofilm growth. After 16 h, biofilms were gently washed with sterile phosphate-buffered saline (PBS) and stained with Cyto-9 (LIVE/DEAD *Bac*Light bacterial viability kit; Thermo Fisher) for 30 min. Biofilms were then washed twice with PBS before imaging using an LSM780 confocal laser scanning microscope (CLSM, Carl Zeiss, Oberkochen, Germany) at 40×. Representative CLSM images from each well were randomly acquired by scanning Z-stacks using ZEN (Zeiss software). All CLSM stacks were processed using Imaris software (Bitplane AG, Zurich, Switzerland) to produce projection images of the biofilms. Biovolumes were extracted from 3-dimensionel (3D) biofilm images using the Imaris (Bitplane AG, Zurich, Switzerland) image analysis package, while surface roughness coefficients were analyzed from 3D confocal images of biofilm using the plug-in COMSTAT 2.1 (78) running in ImageJ (79). A total of 11 images from 3 independent experiments were acquired and processed for each condition.

### Single plane illumination microscopy fluorescence correlation spectroscopy (SPIM-FCS).

The protocol used was as previously described (80). Briefly, SPIM-FCS was used to quantitate the mobility of molecules diffusing in 3D in the biofilm matrix of the P. aeruginosa biofilm.

### Flow cell setup.

The biofilm setup employed is a modification of the conventional flow cell system which consists of a square fluorinated ethylene propylene (FEP) tube (2 cm long, 2.1 mm inner diameter, 2.3 mm outer diameter; Adtech Polymer Engineering Ltd., Aston Down East, Gloucestershire, UK). Flow cells were continuously supplied at a flow speed of 8 ml h^−1^ with minimal medium [15.1 mM (NH_4_)_2_SO_4_, 33.7 mM Na_2_HPO_4_, 22 mM KH_2_PO_4_, 0.05 mM, NaCl, 1 mM MgCl_2_·6H_2_O, 100 μM CaCl_2_·2H_2_O, and 1 μM FeCl_3_·6H_2_0] supplemented with 2 g/liter glucose and 2 g/liter casamino acid. Medium was then supplemented with either testosterone (2.5 μM) or ethanol (control). A 5-ml liquid culture of P. aeruginosa PAO1 was grown overnight at 37°C (200 rpm), and bacterial cultures were diluted in minimal medium to achieve a cell density of 0.2 (OD_600_). Prepared inocula were introduced into the flow cell using a 0.5-ml sterile syringe. The continuous flow was paused to allow bacterial cells to settle and attach to the flow cell. The flow resumed after 1 h, and biofilms were cultured at room temperature for 3 days.

### Preparation of biofilm samples.

FEP tubes containing biofilms were detached from the above-described system, and outlet ends were sealed with silicone glue (3M super silicone clear sealant). Minimal medium supplemented with 100 nM 150 kDa Dextran TRITC (TdB Consultancy, Uppsala, Sweden) was introduced into the tubing containing the biofilms and incubated for 1 h at room temperature. The inlet ends were attached to the SPIM sample capillary.

### SPIM-FCS measurements.

SPIM imaging was carried out using a commercially available light-sheet microscope (Zeiss Lightsheet Z.1; Carl Zeiss AG, Germany). A laser power of 1 to 3 mW was used for illumination. LSFM10x/NA0.2 served as the illumination objective, while a WPlan-Apochromat 63×/NA1.0 was used as the detection objective. Samples were illuminated using a 561-nm laser. The fluorescence was filtered using LP565. Before the region of interest was acquired, the camera was cooled to −80°C, exposure gain was set to 300, and exposure time was set to 0.002 s. A total of 50,000 frames were collected for each sample. The ImFCS plugin (81) was used to compute the diffusion coefficient of the 150-kDa neutral molecule through P. aeruginosa biofilms. The data were binned 3 × 3, and bleach correction was performed using a polynomial of order 4. The model used to fit the data is given below.

$$G\left(\tau \right)=\frac{1}{N}\frac{g\left(\tau \right)}{g\left(0\right)}$$

$$g\left(\tau \right)={\left\{\frac{\sqrt{4D\tau +\omega_{\mathrm{xy}}^{2}}}{a\sqrt{\pi }}\cdot \left[e^{\left(-\frac{a^{2}}{4D\tau +\omega_{\mathrm{xy}}^{2}}\right)}-1\right]+\text{Erf}\left(\frac{a}{\sqrt{4D\tau +\omega_{\mathrm{xy}}^{2}}}\right)\right\}}^{2}\times {\left(1+\frac{4D\tau }{\omega_{Z}^{2}}\right)}^{-\frac{1}{2}}+G_{\infty }$$

where *a* is the pixel size (267 nm), *τ* is the lag time, *N* is the number of particles determined by fitting, *D* is the diffusion coefficient estimated by fitting, ωxy is the *e^−2^* radius in the xy direction (1.5 μm), and ωz is the thickness of the light sheet (1.9 μm).

### Bacterial transcriptomics.

P. aeruginosa PAO1 was cultured overnight in TSB at 37°C under aeration (200 rpm) in the presence of ethanol (control) with or without testosterone (2.5 μM). Then, 1-ml cultures were treated with RNAProtect reagent (Qiagen, Germany) to maintain the integrity of the RNA. Total RNA was then extracted using an RNAeasy minikit (Qiagen, Germany) with some modifications. A Turbo DNA-free kit (Thermo Fisher Scientific, Lithuania) was used to remove genomic DNA contaminants from the total RNA. DNA contamination was assessed with a Qubit double-stranded (dsDNA) high sensitivity assay (PicoGreen dye) and a Qubit 2.0 fluorometer (Invitrogen, Austria) according to the manufacturer’s instructions. rRNA was depleted with a Ribo-Zero rRNA removal kit (Illumina, USA), and integrity of the total RNA was assessed with an Agilent TapeStation system (Agilent Technologies, UK). Double-stranded cDNAs were reverse-transcribed using the NEBNext RNA first- and second-strand synthesis module (NEB, USA). cDNAs were subjected to Illumina’s TruSeq stranded mRNA protocol, and quantitated libraries were pooled at equimolar concentrations and sequenced on an Illumina HiSeq 2500 sequencer in rapid mode at a read length of 100-bp paired ends. Differential expression analysis of RNA-seq data was performed using the Rockhopper analysis suite with default parameters and using P. aeruginosa PAO1 (GenBank accession number NC_002516) and its associated annotation as a reference.

### RNA isolation and Northern blot analysis.

P. aeruginosa PAO1 was grown overnight in TSB liquid culture (180 rpm, 37°C). Bacteria were then diluted 100-fold in 10 ml TSB medium supplemented with 100 μl ethanol (negative vehicle control), testosterone (2.5 μM), estriol (25 nM), or colistin (0.4 μM). Cultures were incubated at 37°C overnight or subcultured three times (100-fold) for a total of 3 days. RNA was isolated using RNeasy Midi prep kits (Qiagen) following the manufacturer’s protocol. The purity and integrity of RNA were determined by spectrophotometry. For Northern blotting, 20 μg of total RNA was separated on an agarose/formaldehyde gel as previously described (82). RNA was then transferred to Hybond N+ membranes (GE Healthcare Life Sciences) by capillary blotting. Membranes were hybridized overnight with ^32^P ɣ-labeled DNA oligonucleotides (Table S2B). Northern blots were developed using a Bio-Rad Molecular Imager FX, and signals were quantified with ImageJ (1.52 h).

### Outer membrane vesicles (OMV).

**Extraction:** The protocol was described previously (83). Briefly, PAO1 was cultivated in TSB (200 rpm, 37°C) with ethanol, ethanol-dissolved testosterone (2.5 μM), estriol (25 nM), or colistin (0.4 μM). Cell-free supernatants were collected by centrifugation of the overnight cultures (10,000 rpm, 10 min, room temperature). Residual cells were removed by filtration of the supernatant through a Nalgene Rapid-Flow sterile disposable filter unit (500 ml) with PES membrane (pore size, 0.2 μm). Filtration was repeated twice to ensure that the supernatants were cell-free. Bacterial MVs were pelleted from filtered supernatants by ultracentrifugation (25,000 rpm, 1.5 to 2 h, 4°C) using a Type 45 Ti rotor. OMVs were then resuspended in 1 ml 1× phosphate buffered saline (PBS; Gibco), at pH 7.

**Purification:** Next, OMVs were purified using density gradient centrifugation (ranging from 35 to 60%) with Opti-Prep (Sigma Aldrich) diluted with 1× PBS. Opti-Prep bands containing OMVs were then washed twice with 1× PBS to remove the Opti-Prep solution.

**Particle count and size measurement:** OMV samples were analyzed using a NanoSight NS300 system to quantify the size and particle concentrations, and the Malvern Zetasizer DLS instrument was used to acquire the hydrodynamic mean vesicle size. Each sample was measured in triplicate, and data were combined for further analysis.

**Proteomic analysis:** Tandem MS was performed by the SciLife facility (Uppsala, Sweden). Briefly, total protein concentration was measured using Bradford protein assays (Pierce kit). For each sample, 15 μg of protein was used for digestion. Proteins were digested with trypsin according to a standard operating procedure. The collected peptides were then purified, dried, and resolved in 0.1% formic acid and further diluted 4 times. Tandem MS was performed by applying high-energy collisional dissociation. Protein identification was based on at least two matching peptides of 95% confidence per protein. Only proteins with a Sequest score above 30 were considered for analysis.

**Transmission electron microscopy (TEM):** Glow-discharged carbon-coated grids (Oxford Instruments, United Kingdom) were incubated for 1 min with a drop of bacterial solution or purified OMVs and negatively stained with 1% uranyl acetate in water. Images were acquired using a high-resolution JEOL ARM300 transmission electron microscope operating at 300 keV with the Gatan one-view CMOS Camera (Gatan, USA).

### Fluorescence recovery after photobleaching (FRAP) assays.

**Cell growth and preparation:** Liquid cultures (2 ml) of P. aeruginosa were cultivated in TSB at 37°C with aeration and shaking (200 rpm). Overnight cultures were washed three times with 1× phosphate-buffered saline (PBS) before cell exposure to a final concentration of 10 μM Nile red dye (Thermo Fisher Scientific). Samples were incubated at room temperature in the dark for 15 to 20 min. Unbound dye was removed following centrifugation (10,000 rpm, 5 min), and bacterial cells were further washed twice with 1× PBS. Then, 10 μl of cells resuspended in PBS was loaded onto a 1% agarose pad (Vivantis LE grade) prepared in 1×X PBS at pH 7.4 (Gibco). Molten agar (500 μl) was sandwiched between two pieces (1 by 3 inches) of microscope glass slides (1 mm to 1.2 mm thickness), and coverslip covering pads were sealed with paraffin wax.

**FRAP measurements:** FRAP measurements were taken with a confocal laser scanning microscope (Carl Zeiss LSM 780) using the 561-nm line at the ×100 lens objective. A rectangular strip with a width of 0.34 μm and length extending across the width of the bacterial cell was drawn in the middle of the cell. The fluorescence within the strip was measured at low laser power (4%) and then photobleached with full laser power (100%) for 0.37 s. Fluorescence recovery was followed immediately with low laser power for ∼5.7 s, the time required to reach a plateau in the fluorescence intensity.

**Data analysis:** Molecular dynamics were semiquantitatively characterized by fitting the FRAP curves to an exponential equation providing the halftime of recovery (t_1/2_) and the mobile fraction measurements. However, our data could not be fitted in a single exponential equation. A bi-exponential equation was, instead, used to fit the curves obtained in our experiments:

$$I\left(t\right)=F_{m1}\left(1-e^{-\tau_{1}t}\right)+F_{m2}\left(1-e^{-\tau_{2}t}\right)$$

where *F*_m1_ and *F*_m2_ represent the mobile fractions. The halftime of recovery was calculated from the rates τ_1_ and τ_2_ obtained from the fit:

$$t_{1/2}=\frac{\ln0.5}{-\tau }$$

whereas the mobile fraction or the fraction of recovery was obtained from

$$F_{m}=F_{m1}+F_{m2}$$

### Animal experiments.

**P. aeruginosa** ***infection*:** Specific-pathogen-free, 6- to 10-week-old, female BALB/c mice were obtained from the University of Newcastle (New South Wales, Australia) central animal house. Animals were housed on a 12-h light:dark cycle in individually ventilated cages with food and water available *ad libitum*. All experimental procedures were performed under aseptic techniques and with approval from the University of Newcastle Animal Care and Ethics Committee.

P. aeruginosa (PAO1) was grown overnight (37°C, shaking) in Luria-Bertani (LB) broth supplemented with testosterone (2.5 μM; Sigma-Aldrich, Castle Hill, Australia), estriol (25 nM; Sigma-Aldrich), or ethanol (Sigma-Aldrich). Bacteria were quantified by spectrophotometry (600 nM; iMark microplate reader; Bio-Rad, Hercules, CA, USA) and regression curve analysis relating known values to optical density. Animals were intranasally infected with 10^6^ CFU of pretreated PAO1 in 30 μl of PBS under light isoflurane anesthesia. Animals were then euthanized by lethal intraperitoneal injection of sodium pentobarbitone at 0.5 and 6 h postinfection. BALF was then collected from the airways by lavaging the lungs twice with 0.5 ml of PBS. Lavaged lung tissue was then collected into 1 ml of PBS and homogenized on ice. BALF and homogenized lung tissue were plated onto LB agar plates (20 μl, 5 × 1:10 serial dilutions) and incubated overnight (37°C, 24 h), and the CFU were counted to determine bacterial titers in tissues. Results are presented as combined BALF and lung bacterial titers and those from individual tissues (84–86). Cells were collected from the BALF (295 × *g*, 4°C, 7 min), with red blood cells lysed (Tris-buffered NH_4_Cl, 500 μl, 5 min). The remaining leukocytes were collected (295 × *g*, 4°C, 7 min) and counted using a hemocytometer. The leukocytes were cytospun (300 rpm, 10 min; Thermo Fisher Scientific, Scoreby, Australia) onto microscope slides, air-fixed, and then stained with May-Grunwald-Giemsa. Differential counts of inflammatory cells were performed under light microscopy (×40 magnification) and determined by size and morphology (84).

**OMV *in vivo* challenge:** For OMV experiments, all experimental procedures were performed under aseptic techniques and with approval from the IACUC committee of the Shenzhen Institute of Advanced Technology, Chinese Academy of Sciences. C57BL/6 mice (female, 6 to 8 weeks old) underwent intratracheal installation of OMV extracts in 70 μl sterile saline using a standardized concentration of a total of 10 μg protein. Twenty-four hours after OMV administration, BALF was harvested by flushing 1 ml of sterile saline into the lungs through a catheter inserted into the mouse trachea. Injected saline was retracted with a 3-ml syringe, centrifuged at 3,500 rpm, and resuspended in red blood cell lysis buffer (Beyotime; C3702). Supernatant from the saline centrifugation was collected for Luminex multicytokine analysis using a Bioplex 23-cytokine mouse panel (Bio-Rad; M60009RDPD). The lung tissues were then dissected and fixed in 4% paraformaldehyde before serial dehydration in ethanol, xylene, and paraffin. Paraffin blocks were sectioned at 5 μm, stained for hematoxylin and eosin, and processed for histology.

### Statistical analysis.

Unless stated otherwise in the experimental procedures, statistical significance was determined by one-way analysis of variance (ANOVA) corrected using Dunnett’s test for multiple comparisons. Differences were considered statistically significant when the *P* values were <0.05. Analysis was performed using GraphPad Prism 7.0.

### Data availability.

The MS proteomics data related to potential steroid-binding proteins were deposited in the ProteomeXchange Consortium via the PRIDE (87) partner repository with the data set identifiers PXD018897 and 10.6019/PXD018897. Reviewer account details are as follows: username, reviewer96473@ebi.ac.uk; password, kQhOxvyM. The MS proteomics data related to OMVs were deposited into the ProteomeXchange Consortium via the PRIDE (87) partner repository with the data set identifier PXD019470. Reviewer account details are as follows: username, reviewer94411@ebi.ac.uk; password, kl78AUPh. The transcriptomics data discussed above have been deposited in NCBI’s Gene Expression Omnibus (88) and are accessible through the GEO Series accession number GSE148955. These 3 data sets were used to generate transcriptomic data (Fig. 2A) and proteomic data for steroid protein binders (Table S1A) and the OMV protein profile (Table S1B).

10.1128/mBio.01774-20.1 TEXT S1 Supplemental methods. Download Text S1, DOCX file, 0.03 MB. Copyright © 2020 Vidaillac et al. 2020 Vidaillac et al. This content is distributed under the terms of the Creative Commons Attribution 4.0 International license.
