# Erratum for Vidaillac et al. “Sex Steroids Induce Membrane Stress Responses and Virulence Properties in Pseudomonas aeruginosa”

**DOI:** 10.1128/mBio.02809-20

**Published:** 2020-11-03

**Authors:** Celine Vidaillac, Valerie Fei Lee Yong, Marie-Stephanie Aschtgen, Jing Qu, Shuowei Yang, Guangfu Xu, Zi Jing Seng, Alexandra C. Brown, Md Khadem Ali, Tavleen K. Jaggi, Jagadish Sankaran, Yong Hwee Foo, Francesco Righetti, Anu Maashaa Nedumaran, Micheál Mac Aogáin, Dan Roizman, Jean-Alexandre Richard, Thomas R. Rogers, Masanori Toyofuku, Dahai Luo, Edmund Loh, Thorsten Wohland, Bertrand Czarny, Jay C. Horvat, Philip M. Hansbro, Liang Yang, Liang Li, Staffan Normark, Birgitta Henriques-Normark, Sanjay H. Chotirmall

**Affiliations:** a Lee Kong Chian School of Medicine, Nanyang Technological University, Singapore; b Singapore Centre for Environmental Life Sciences Engineering, Nanyang Technological University, Singapore; c Department of Microbiology, Tumor and Cell Biology, Karolinska Institutet, Stockholm, Sweden; d Karolinska University Hospital Solna, Stockholm, Sweden; e Shenzhen Institutes of Advanced Technology, Chinese Academy of Sciences, Shenzhen, Guangdong, China; f Department of Otolaryngology, The Fifth Affiliated Hospital of Sun Yat-Sen University, Zhuhai, Guangdong, China; g Priority Research Centre for Healthy Lungs, Hunter Medical Research Institute, Newcastle, New South Wales, Australia; h School of Biomedical Sciences and Pharmacy, University of Newcastle, Newcastle, New South Wales, Australia; i Department of Biological Sciences, National University of Singapore, Singapore; j Centre for Bio-Imaging Sciences, National University of Singapore, Singapore; k School of Materials Science and Engineering (MSE), Nanyang Technological University, Singapore; l Functional Molecules and Polymers, Institute of Chemical and Engineering Sciences, ICES, Agency for Science, Technology, and Research (A*STAR), Singapore; m Department of Clinical Microbiology, Trinity College Dublin, St. James’s Hospital Campus, Dublin, Ireland; n Faculty of Life and Environmental Sciences, University of Tsukuba, Tsukuba, Japan; o Centre for Inflammation, Centenary Institute, Sydney, NSW, Australia; p University of Technology Sydney, School of Life Sciences, Faculty of Science, Ultimo, NSW, Australia

## ERRATUM

Volume 11, no. 5, e01774-20, 2020, https://doi.org/10.1128/mBio.01774-20. Professor Birgitta Henriques-Normark’s last name was missing a hyphen between Henriques and Normark in the original article. The byline should appear as shown above. Also, the affiliations and affiliation letters are correctly represented herein.

