# Supplementary material for: Sex Steroids Induce Membrane Stress Responses and Virulence Properties in Pseudomonas aeruginosa
Source: mBio. 2020 Sep 29;11(5):e01774-20. doi: 10.1128/mBio.01774-20 (PMC7527723; doi:10.1128/mBio.01774-20)
Supplement: TABLE S1 [file mBio.01774-20-st001.docx]

**Table S1A**

| **GENE SYMBOL** | **MOLECULAR WEIGHT** | **COVERAGE** | **HORMONE TREATMENT** |  |
| --- | --- | --- | --- | --- |
|  |  |  |  |  |
| PA3515 | 39.74 | 3.1 | Testosterone |  |
| PA2734 | 48.71 | 6.5 | Testosterone |  |
| Vfr | 24.21 | 21.5 | Testosterone |  |
| gpmI | 55.57 | 12.2 | Testosterone |  |
| nuoB | 25.41 | 13.8 | Testosterone |  |
| PA1746 | 18.18 | 16.1 | Testosterone |  |
| PA4336 | 21.01 | 10.8 | Testosterone |  |
| PA4673.10 | 15.42 | 25 | Testosterone |  |
| Hom | 46.2 | 8.3 | Testosterone |  |
| PA4917 | 22.44 | 7.5 | Testosterone |  |
| hisH1 | 23.68 | 5.2 | Testosterone |  |
| PA1009 | 20.57 | 5.4 | Testosterone |  |
| ureC | 60.57 | 3.7 | Testosterone |  |
| dctB | 68.49 | 0.655 | Estradiol |  |
| PA2839 | 28.14 | 0.633 | Estradiol |  |
| PA0622 | 41.18 | 0.606 | Estradiol |  |
| PA3127 | 29.39 | 0.596 | Estradiol |  |
| bamD | 38.59 | 0.584 | Estradiol |  |
| PA3035 | 22.38 | 0.576 | Estradiol |  |
| PA0058 | 24.91 | 0.573 | Estradiol |  |
| Aat | 25.81 | 0.566 | Estradiol |  |
| Nth | 23.72 | 0.505 | Estradiol |  |
| PA0078 | 48.94 | 0.488 | Estradiol |  |
| PA0285 | 86.33 | 0.483 | Estradiol |  |
| aprE | 48.02 | 0.481 | Estradiol |  |
| azoR3 | 23.88 | 0.479 | Estradiol |  |
| PA4621 | 103.7 | 0.474 | Estradiol |  |
| PA4734 | 32.63 | 0.474 | Estradiol |  |
| PA1335 | 46.68 | 0.468 | Estradiol |  |
| PA5310 | 59.16 | 0.442 | Estradiol |  |
| rmuC | 51.51 | 0.442 | Estradiol |  |
| PA4636 | 43.75 | 0.435 | Estradiol |  |
| PA3191 | 53.02 | 0.429 | Estradiol |  |
| flgI | 38.16 | 0.428 | Estradiol |  |
| mucB | 34.55 | 0.421 | Estradiol |  |
| flhA | 75.83 | 0.405 | Estradiol |  |
| PA5133 | 47.26 | 0.4 | Estradiol |  |
| ndvB | 96.9 | 0.391 | Estradiol |  |
| PA3883 | 29.41 | 0.391 | Estradiol |  |
| PA4358 | 82.46 | 0.363 | Estradiol |  |
| Ppc | 97.78 | 0.343 | Estradiol |  |
| PA1814 | 28.41 | 0.336 | Estradiol |  |
| PA3024 | 57.98 | 0.335 | Estradiol |  |
| atuD | 42.69 | 0.334 | Estradiol |  |
| PA5362 | 49.95 | 0.325 | Estradiol |  |
| PA5307 | 92.9 | 0.325 | Estradiol |  |
| PA1341 | 27.58 | 0.302 | Estradiol |  |
| narX | 69.28 | 0.302 | Estradiol |  |
| PA4619 | 44.68 | 0.301 | Estradiol |  |
| PA2542 | 130.48 | 0.293 | Estradiol |  |
| PA2222 | 23.43 | 0.293 | Estradiol |  |
| lpxA | 27.99 | 0.267 | Estradiol |  |
| PA0860 | 65.42 | 0.26 | Estradiol |  |
| PA4918 | 23.81 | 0.247 | Estradiol |  |
| yidC | 64.03 | 0.246 | Estradiol |  |
| opgH | 96.93 | 0.224 | Estradiol |  |
| PA5291 | 73.41 | 0.163 | Estradiol |  |
| PA3211 | 40.67 | 0.155 | Estradiol |  |
| putP | 54.38 | 0.115 | Estradiol |  |
| PA1819 | 47.74 | 0.102 | Estradiol |  |
| fruA | 58.95 | 0.101 | Estradiol |  |
| PA0756 | 24.42 | 4 | Estradiol |  |
| PA3336 | 39.51 | 2.3 | Estradiol |  |

**Table S1B**

| **ACCESSION NUMBER** | **GENE SYMBOL** | **DESCRIPTION** | **FUNCTION** | **LOCATION** | **COVERAGE** | |
| --- | --- | --- | --- | --- | --- | --- |
|  |  |  |  |  | **Control** | **Hormones** |
| A0A3G5V5J7 | - | OmpA family protein | Transporter | OM | Ethanol (44.01) | E3 (111.07) /T (85.75) |
| P33641 | PA4545 | Outer membrane protein assembly factor BamD | Cell envelope | OM | - | E3 (31.16) |
| G3XD83 | PA0625 | Uncharacterized protein | Hypothetical protein | OM | - | E3 (33.56) /T (51.13) |
| A0A3P3P0F7 | OpmH | Efflux transporter outer membrane subunit OpmH | Transporter | OM | - | E3 (35.92) |
| A0A367M0Q0 | TaqQ | Type VI secretion-associated lipoprotein TagQ (Fragment) | Toxin/Lipoprotein | OM | - | E3 (36.35) |
| A0A2U9EWV7 | oprD | OprD | Transporter | OM | - | E3 (37.31) /T (38.64) |
| A0A3G5EJT7 | oprM | Outer membrane channel subunit OprM | Transporter | OM | - | E3 (37.95) |
| A6V9E3 | - | Lipoprotein, putative | Enzymatic | OM | - | E3 (39.01) |
| A0A1C7BSZ3 | - | Metalloproteinase outer membrane | Unknown | OM | - | E3 (40.01) |
| A0A0H2ZGY2 | - | Putative lipoprotein | Enzymatic | OM | - | E3 (40.98) |
| P11221 | oprI | Major outer membrane lipoprotein | Enzymatic | OM | - | E3 (41.87) |
| A0A3P3NJ14 | - | LPS-assembly protein LptD | Cell envelope | OM | - | E3 (55.94) /T (34.67) |
| A0A3P3I0D6 | - | OprD family porin | Transporter | OM | - | E3 (58.01) /T (43.44) |
| A0A367MCG2 | - | TonB-dependent copper receptor | Other | OM | - | E3 (58.85) /T (38.34) |
| A0A1G9YUD9 | - | OmpA-OmpF porin | Transporter | OM | - | E3 (61.93) |
| A0A3M5D3Q1 | - | Uncharacterized protein | Cell envelope | OM | - | E3 (65.71) /T (41.42) |
| Q9I120 | PA2462 | Uncharacterized protein | Enzymatic | OM | - | E3 (110.52) /T (105.85) |
| A0A3P3I4T6 | - | OprD family porin | Transporter | OM | - | T (30.07) |
| A0A0A8RHX3 | - | Lipoprotein NlpD/LppB homolog | Other | OM | - | T (30.94) |
| A0A3G5V5J7 | - | OmpA family protein | Transporter | OM | - | T (85.75) |
| A0A1C7BNL1 | - | Flagellin | Motility | EC | Ethanol (171.53) | - |
| A0A0A8RPG4 | - | Uncharacterized protein | Other | EC | - | T (40.61) |
| A0A2V3GTP2 | - | Tol-Pal system protein TolB (Fragment) | Transporter | P | - | T (34.35) |
| A0A0G6CQ56 | - | 60 kDa chaperonin | Other | C | Ethanol (30.48) | - |
| A0A367MAG8 | GroL | 60 kDa chaperonin (Fragment) | Protein folding | C | Ethanol (54.12) | - |
| A6UZJ1 | RplB | 50S ribosomal protein L2 | Other | C | - | T (32.97) |
| A6VF32 | AtpD | ATP synthase subunit beta | Enzymatic | C | - | T (38.44) |
| A0A3P3HGP2 | - | DNA-directed RNA polymerase subunit beta | Enzymatic | C | - | T (38.87) |
| A0A127N2P4 | AtpA | ATP synthase subunit alpha | Enzymatic | C | - | T (51.27) |
| A0A0H2ZID8 | AceF | Acetyltransferase component of pyruvate dehydrogenase complex | Metabolism | C | - | T (58.75) |
| A0A1J0J186 | AceE | Pyruvate dehydrogenase E1 component | Enzymatic | C | - | T (63.59) |
| A0A386YWU8 | - | LysM peptidoglycan-binding domain-containing protein | Unknown | U | - | T (34.03) |
| A0A3M5EB07 | - | Uncharacterized protein (Fragment) | Unknown | U | - | T (36.80) |
| A0A1C7BUG8 | - | Uncharacterized protein | Unknown | U | - | T (61.69) |
