# Supplementary material for: Sex Steroids Induce Membrane Stress Responses and Virulence Properties in Pseudomonas aeruginosa
Source: mBio. 2020 Sep 29;11(5):e01774-20. doi: 10.1128/mBio.01774-20 (PMC7527723; doi:10.1128/mBio.01774-20)
Supplement: TABLE S2 [file mBio.01774-20-st002.docx]

**Table S2A**

| **Patient number** | **Specimen Site (Type)** | **Year of isolate** | **Age of patient (Years)** | **Gender** | **Primary site of infection** | **HAI** | **ICU**  **admission** | **Known antibiotic resistance** | **Known antibiotic susceptibility** |
| --- | --- | --- | --- | --- | --- | --- | --- | --- | --- |
| 1 | Urine | 2016 | 80 | M | Urine | No | No |  | PIP, TZP, CIP, GEN |
| 2 | Blood | 2014 | 82 | M | Systemic | No | Yes |  | PIP, TZP, CIP, GEN |
| 3 | Swab | 2016 | 81 | F | Lower leg | No | No |  | PIP, TZP, CIP, GEN |
| 4 | Blood | 2015 | 70 | F | Systemic | No | Yes | IMP, MEM (I) | PIP, TZP, CAZ, CIP, AMK, GEN |
| 5 | Sputum | 2016 | 86 | F | Lung | No | No |  | PIP, TZP, CIP, GEN |
| 6 | Blood | 2015 | 60 | F | Systemic | Yes | Yes | PIP, TZP, CAZ | IMP, MEM, CIP, AMK, GEN |
| 7 | Blood | 2015 | 60 | M | Systemic | Yes | No |  | IMP, MEM, CIP, AMK, GEN, PIP, TZP, CAZ |
| 8 | Swab | 2016 | 71 | M | Right toe | No | No |  | PIP, TZP, CIP, GEN |
| 9 | Blood | 2015 | 50 | F | Systemic | Yes | No |  | IMP, MEM, CIP, AMK, GEN, PIP, TZP, CAZ |

HAI: Hospital Acquired Infection, ICU: Intensive Care Unit, M: Male, F: Female, PIP: piperacillin, TZP: Tazobactam, CAZ: Ceftazidime, IMP: imipenem, MEM: Meropenem, CIP: Ciprofloxacin, AMK: Amikacin, GEN: Gentamycin, I: Intermediate susceptibility

**Table S2B**

| **STRAIN** | **DESCRIPTION** | **SOURCE** | **CITATION** |
| --- | --- | --- | --- |
| PAO1 | Prototypic nonmucoid wild-type strain | SCELSE, NTU | Chua et al., *Nat Commun* **5**, 4462 (2014). |
| PAO1 | Wild type | University of Tsukuba, Japan | Tashiro et al., *J Bacteriol* **191**, 7509-7519 (2009). |
| Δ*mucB* mutant | PAO1Δ*mucB* mutant | University of Tsukuba, Japan | Tashiro et al., *J Bacteriol* **191**, 7509-7519 (2009). |
| Δ*mucB:mucB* mutant | PAO1Δ*mucB:mucB mutant* | LKC, NTU, Singapore | This study |
| PAO1 | Wild type, Two-Allele Library | University of Washington, Seattle, WA, USA | Jacobs et al., *Proc Natl Acad Sci U S A* **100**, 14339-14344 (2003). |
| Δ*mucA* mutant | *mucA*-A05::ISphoA/hah, Two-Allele Library | University of Washington, Seattle, WA, USA | Jacobs et al., *Proc Natl Acad Sci U S A* **100**, 14339-14344 (2003). |
| Δ*algR* mutant | *algR*-C11::ISphoA/hah, Two-Allele Library | University of Washington, Seattle, WA, USA | Jacobs et al., *Proc Natl Acad Sci U S A* **100**, 14339-14344 (2003). |
| Δ*vfr* mutant | PAO1Δ*vfr* mutant | SCELSE, NTU, Singapore | Zhang et al., Front Cell Infect Microbiol 9, 164 (2019). |
| Δ*vfr:vfr* mutant | PAO1Δ*vfr:vfr* mutant | LKC, NTU, Singapore | NA |
| **PLASMIDS** | **DESCRIPTION** | **SOURCE** | **CITATION** |
| pUC18 | Ap^r^ | Bio Basic Inc. | This study |
| pUC*mucB* | pUC18 with an entire fragment of *mucB* between *Sal*I and *Hin*dIII; Ap^r^ | Bio Basic Inc. | This study |
| pUC*vfr* | pUC18 with an entire fragment of *vfr* between *Sal*I and *Hin*dIII; Ap^r^ | Bio Basic Inc. | This study |
| **PRIMERS** | **SEQUENCES** | **SOURCE** | **CITATION** |
| *mucA* | ATGACGGACCGCGCCAACTGGTAACGCGACCAGGTGGAACGCAGCTCGGC | Integrated DNA Technologies (IDT) | This study |
| *pilA* | GTCTTCAGCGGGTGATCGTCGCCAGCGCCGAAGCACCTTCCGAACGCGC | Integrated DNA Technologies (IDT) | This study |
| *16sRNA* | CAAGTCGAGCGATGAAGGGAGCTTGCTCCTGGATTCAGCGGCGGACGGG | Integrated DNA Technologies (IDT) | This study |
| vfr_F | TATTTCTCCGCCGAGCTC | Integrated DNA Technologies (IDT) | This study |
| vfr_R | GAAACCACCCTCGACCTGTT | Integrated DNA Technologies (IDT) | This study |
| vfr_UpF | AGCTCTACCCGGGCGCCTTCTTCAGGATGCTGA | Integrated DNA Technologies (IDT) | This study |
| vfr_UpR | CACCTTGCGTGAGGTCTT | Integrated DNA Technologies (IDT) | This study |
| vfr_DownF | CGACGGCCAGTGCCAGGTCACCGAGAGCGGTATTC | Integrated DNA Technologies (IDT) | This study |
| vfr_DownR | CGACGGCCAGTGCCAGGTCACCGAGAGCGGTATTC | Integrated DNA Technologies (IDT) | This study |
