# Supplementary material for: Sex Steroids Induce Membrane Stress Responses and Virulence Properties in Pseudomonas aeruginosa
Source: mBio. 2020 Sep 29;11(5):e01774-20. doi: 10.1128/mBio.01774-20 (PMC7527723; doi:10.1128/mBio.01774-20)
Supplement: TEXT S1 [file mBio.01774-20-s0001.docx]

Supplementary Information for:

Sex steroids induce membrane stress responses and virulence properties in

*Pseudomonas aeruginosa*

Celine Vidaillac,^a,b*^ Valerie Fei Lee Yong,^a^ Marie-Stephanie Aschtgen,^a,b,c^ Jing Qu,^d^ Shuowei Yang,^e^ Guangfu Xu,^e^ Zi Jing Seng,^b*^ Alexandra C. Brown,^f^ Md Khadem Ali,^f*^ Tavleen K. Jaggi,^a^ Jagadish Sankaran,^g,h*^ Yong Hwee Foo,^b^ Francesco Righetti,^c^

Anu Maashaa Nedumaran,^i^ Micheál Mac Aogáin,^a*^ Dan Roizman,^b^ Jean-Alexandre

Richard,^j^ Thomas R. Rogers,^k^ Masanori Toyofuku,^l^ Dahai Luo,^a^ Edmund Loh,^b,c^ Thorsten Wohland,^g,h^ Bertrand Czarny,^a,i^ Jay C. Horvat,^f^ Philip M. Hansbro,^f,m^

Liang Yang,^b*^ Liang Li ^d^ Staffan Normark,^a,b,c^ Birgitta Henriques Normark,^a,b,c^ and

Sanjay H. Chotirmall^a#^

**This file includes:**

Supplementary Methods

**SUPPLEMENTARY METHODS**

**Synthesis of Testosterone WATER SOLUBLE DERIVATIVE TSDG**

All reactions were carried out under argon atmosphere with dry solvents under anhydrous conditions. Reagents were purchased at the highest commercial quality and used without further purification. Methanol (CH_3_OH) was purchased in anhydrous form and used without further purification. Water, ethyl acetate (EtOAc), diethyl ether (Et_2_O), and methylene chloride (CH_2_Cl_2_) were purchased at the highest commercial quality and used without further purification. Yields refer to chromatographically and spectroscopically (^1^H NMR) homogeneous materials. Reactions were monitored by thin-layer chromatography (TLC) carried out on 0.25 mm E. Merck silica gel plates (60F-254) using UV light as visualizing agent and a solution of potassium permanganate and heat as developing agents. E. Merck silica gel (60, particle size 0.040−0.063 mm) was used for flash column chromatography.

# Instruments and methods

^1^H- and ^13^C-NMR spectra were recorded on a Bruker DRX-400 (400 MHz) instrument. Chemical shifts are expressed in parts per million (ppm) from the residual non-deuterated solvent signal. *J* values are expressed in Hz and the following abbreviations were used to explain the multiplicities: s = singlet, d = doublet, t = triplet, q = quartet, quint = quintet, m = multiplet, pent = pentet, hex = hexet, br = broad.

**Synthesis of Testosterone 17β-*N*,*N*-dimethylglycinate (TSDG) (Supplementary figure 8C)**

To a solution of testosterone (100 mg, 0.35 mmol) in CH_2_Cl_2_ at 25°C were added *N*,*N*-dimethylglycine hydrochloride (59 mg, 0.42 mmol), 1-ethyl-3-(3-dimethylaminopropyl)carbodiimide (74 mg, 0.39 mmol) and 4-dimethylaminopyridine (4 mg, 0.035 mmol) and the medium was stirred at 25 °C for 16 hours. Deionized water and CH_2_Cl_2_ (10 mL each) were added, the organic layer was collected and the aqueous layer extracted once with CH_2_Cl_2_ (10 mL) The combined organic layer was dried on Na_2_SO_4_, filtered and concentrated. Purification on silica (CH_2_Cl_2_/AcOEt : 1/1 to recover the remaining testosterone then CH_2_Cl_2_ / CH_3_OH : 95/5) provided TSDG as a colorless oil (47 mg; 36%).

**TSDG**: ^1^H NMR (400 MHz, CDCl_3_) δ 5.69 (s, 1H), 4.65 (dd, *J* = 9.2, 7.8 Hz, 1H), 3.14 (s, 2H), 2.42–2.29 (m, 9H), 2.26–2.13 (m, 2H), 2.01–1.96 (m, 1H), 1.86–1.74 (m, 2H), 1.73–1.61 (m, 2H), 1.60–1.49 (m, 3H), 1.43–1.28 (m, 2H), 1.24–1.20 (m, 1H), 1.18–1.14 (s, 3H), 1.09–0.89 (m. 3H), 0.82 (s, 3H); ^13^C NMR (100 MHz, CDCl_3_) δ = 199.3, 170.8, 170.6, 123.9, 82.5, 60.3, 53.7, 50.2, 45.2 (2C), 42.5, 38.6, 36.6, 35.7, 35.4, 33.9, 32.7, 31.5, 27.5, 23.4, 20.5, 17.4, 12.1.(**Fig S7C**)

**Synthesis of Testosterone 17β-*N*,*N*-dimethylglycinate hydrochloride (TSDG • HCl) (Fig S7A):**

To a solution of TSDG (47 mg, 0.13 mmol) in ether (2 mL in a 4 mL vial) was added HCl (2 M in ether, 100 µL, 0.20 mmol). A white solid instantly formed, the ether was removed under vacuum and the solid was further dried to give 53 mg of a white powder (quantitative yield).

**TSDG • HCl**: ^1^H NMR (400 MHz, CDCl_3_) δ 13.12 (s, 1H), 5.72 (s, 1H), 4.70 (dd, *J* = 9.2, 7.6 Hz, 1H), 3.92 (s, 2H), 3.01 (s, 6H), 2.48–2.15 (m, 6H), 2.06–1.97 (m, 1H), 1.91–1.76 (m, 2H), 1.73–1.53 (m, 5H), 1.46–1.33 (m, 2H), 1.18 (s, 3H), 1.11–0.90 (m, 3H), 0.84 (s, 3H); ^13^C NMR (100 MHz, CDCl_3_) δ = 199.4, 170.6, 164.7, 124.2, 85.3, 66.0, 55.6, 53.7, 50.2, 42.9, 42.6, 38.7, 36.8, 35.8, 35.5, 34.0, 32.7, 31.5, 27.5, 23.6, 20.6, 17.5, 12.3.(**Fig S7D**)

**Growth curves experiments**

Cultures of *P. aeruginosa* strain PAO1 were prepared in tryptic soy broth supplemented with ethanol (control) or steroids at optimal concentrations to induce virulence. Growth assays were performed in clear flat-bottom BD Falcon 8-well plates (BD Biosciences, San Jose, CA) containing 2 ml of culture per condition tested. Plates were incubated at 37°C in a Tecan Infinite M1000 plate reader (Tecan US Inc., Durham, NC) set with orbital shaking of 2 mm amplitude. Cell density (OD_600_) was measured every hour for the duration of the assay (18 hours). Experiment was done in triplicate to ensure experimental reproducibility.

**Protein purification**

Overexpression of the his-tagged *mucB, mucA* peri and *vfr* were carried out in chemical-competent BL21 (DE3) *E. coli* and was induced with 1mM Isopropyl β- d-1-thiogalactopyranoside (IPTG) 16-20 hours at 18°C. Bacterial pellets were resuspended in lysis buffer (20 mM Imidazole, 50 mM Tris-HCL pH 7.5, 300 mM NaCl, 4 mM 2-Mercaptoethanol, 10% Glycerol) with cOmplete™, Mini, EDTA-free Protease Inhibitor Cocktail (Merck). Cells were lysed by sonication and after centrifugation resuspended pellet through the cooled and equilibrated Nano DeBEE High-Pressure Homogenizer between 800 – 1000 bar pressure for 4 – 6 rounds to achieve a homogenized cell lysate. After centrifugation at 40,000 rcf for 40 minutes, the supernatant was collected and filtered with 0.2 μm membrane filter (Pall corporation) to run through the equilibrated his-tag purification system (ÄKTA start). His-tagged proteins were then eluted with buffer containing a higher concentration of imidazole (500 mM Imidazole, 50 mM Tris-HCL pH 7.5, 300 mM NaCl, 4 mM 2-Mercaptoethanol, 10% Glycerol). Proteins were then dialyzed overnight at 4°C to remove imidazole. Proteins were further purified by HiLoad Superdex 75 pg prepacked columns using size exclusion buffer (150mM NaCl, 25 mM HEPES pH 7.5, 2 mM DTT). Finally, SDS-PAGE gel was run to analyze the protein peak to determine their relative purity. Proteins were diluted to concentration of 1mg/ml and snap freeze with liquid nitrogen and store at -80°C until use.

**CO-IMMUNOPRECIPITATION AND MASS SPECTROSCOPY (MS)**

*P. aeruginosa* PAO1 was cultured in TSB supplemented with ethanol, estradiol or testosterone for three days. Cultures (1 L) were centrifuged at 6,000rpm for 20 minutes. A lysis buffer containing 20mM Tris-HCl (pH 7.6), 150mM NaCl, 0.5% Triton X-100, 0.5 mg/mL lysozyme, Protease inhibitor cocktail (1X) and 10% Glycerol were added to each bacterial pellet and mixed for 1 hour at 4°C on a rocker. Samples were then sonicated three times for 30 seconds each at 90% duty using a Sonics vibra cell sonicator and centrifuged at 10,000rpm for 20 minutes before isolating total protein extract. After pre-clearing the samples with protein-G control beads (Cat.No: 11243233001, Roche) for 1 hour, 5mg of total protein was then added to 100 µL of estradiol beads (Cat.No: 24861-1, Polysciences, Inc.) or protein-G control beads (Cat.No: 11243233001, Roche). Proteins were allowed to bind to the beads overnight at 4°C on a roller after which beads were washed at 4°C four times for 5 minutes each using lysis buffer to remove non-specific interactions. For the isolation of Testosterone binding proteins, an anti-testosterone antibody (14P1F9 Abcam) was used to overcome the absence of a commercially available bead system. After pre-clearing with protein-G beads, 5mg of total protein was bound to the antibodies overnight at 4°C and total protein extract unexposed to antibodies serving as a control for the co-immunoprecipitation. Samples were next added to protein-G beads and allowed to bind for 1 hour at 4°C to capture antibody-protein complexes. Bound proteins were then eluted with 2X laemmli sample buffer with 5% mercaptoethanol. The eluted proteins were run on a 10% SDS-PAGE and the gel lanes excised and sent to the Taplin biological mass spectrometry facility at Harvard University for protein identification using LC-MS/MS.

**SURFACE PLASMON RESISTANCE (SPR)**

Immobilisation of the *P. aeruginosa* purified proteins: Vfr and MucB were performed on carboxymethyl dextran-coated sensor chips CM5 by carbodiimide covalent linkage following the manufacturer’s instructions (Amine Coupling Kit, Biacore AB). Before immobilization, Vfr and MucB were concentrated at 1mg/ml using dialysis against 150mM NaCl, 25 mM HEPES pH 7.5, 2mM DTT (1X running buffer). Vfr and MucB were then diluted more than 10-fold using 10 mM sodium acetate buffer at pH 5.0. Interaction of Vfr and MucB with the respective hormones (testosterone or estriol) was assessed using SPR analysis. Purified protein MucA was used as a positive control for the interaction with MucB, while cAMP used as a positive control for interaction with Vfr. During the analysis of the respective hormone’s interaction with immobilized MucB or Vfr surfaces, running buffer was changed to 1X running with 5% Dimethyl sulfoxide (DMSO; Sigma Aldrich, used as a solvent for hormones). DMSO calibration buffer was made from 1.05X running buffer that was used for DMSO solvent correction before running hormones through the surface. Hormones were serially diluted 2-fold from 200 µM for Vfr and 10 µM for MucB with 1.05X running buffer for the first dilution and 1X with 5% running buffer for subsequent dilutions. The dissociation rate (kd) constant was determined through a nonlinear curve fitting using the Pharmacia Biosensor kinetics software BIA evaluation 2.1 (Biacore AB, Pharmacia).

**SUPPLEMENTARY REFERENCES**

1. Chua SL*, et al.* Dispersed cells represent a distinct stage in the transition from bacterial biofilm to planktonic lifestyles. *Nat Commun* **5**, 4462 (2014).
2. Tashiro Y*, et al.* Outer membrane machinery and alginate synthesis regulators control membrane vesicle production in Pseudomonas aeruginosa. *J Bacteriol* **191**, 7509-7519 (2009).
3. Jacobs MA*, et al.* Comprehensive transposon mutant library of Pseudomonas aeruginosa. *Proc Natl Acad Sci U S A* **100**, 14339-14344 (2003).
4. Zhang Y, et al. Glutathione Activates Type III Secretion System Through Vfr in Pseudomonas aeruginosa. Front Cell Infect Microbiol 9, 164 (2019).
